# The genome-wide role of HSF-1 in the regulation of gene expression in *Caenorhabditis elegans*

**DOI:** 10.1186/s12864-016-2837-5

**Published:** 2016-08-05

**Authors:** Jessica Brunquell, Stephanie Morris, Yin Lu, Feng Cheng, Sandy D. Westerheide

**Affiliations:** 1 Department of Cell Biology, Microbiology, and Molecular Biology, College of Arts and Sciences, University of South Florida, Tampa, FL 33620 USA; 2 Department of Pharmaceutical Sciences, College of Pharmacy, University of South Florida, Tampa, FL 33612 USA; 3 Department of Epidemiology and Biostatistics, College of Public Health , University of South Florida, Tampa, FL 33620 USA

**Keywords:** RNA-seq, Heat shock response, Stress, *C. elegans*, Transcript analysis, HSF-1

## Abstract

**Background:**

The heat shock response, induced by cytoplasmic proteotoxic stress, is one of the most highly conserved transcriptional responses. This response, driven by the heat shock transcription factor HSF1, restores proteostasis through the induction of molecular chaperones and other genes. In addition to stress-dependent functions, HSF1 has also been implicated in various stress-independent functions. In *C. elegans*, the HSF1 homolog HSF-1 is an essential protein that is required to mount a stress-dependent response, as well as to coordinate various stress-independent processes including development, metabolism, and the regulation of lifespan. In this work, we have performed RNA-sequencing for *C. elegans* cultured in the presence and absence of *hsf-1* RNAi followed by treatment with or without heat shock. This experimental design thus allows for the determination of both heat shock-dependent and -independent biological targets of HSF-1 on a genome-wide level.

**Results:**

Our results confirm that *C. elegans* HSF-1 can regulate gene expression in both a stress-dependent and -independent fashion. Almost all genes regulated by HS require HSF-1, reinforcing the central role of this transcription factor in the response to heat stress. As expected, major categories of HSF-1-regulated genes include cytoprotection, development, metabolism, and aging. Within both the heat stress-dependent and -independent gene groups, significant numbers of genes are upregulated as well as downregulated, demonstrating that HSF-1 can both activate and repress gene expression either directly or indirectly. Surprisingly, the cellular process most highly regulated by HSF-1, both with and without heat stress, is cuticle structure. Via network analyses, we identify a nuclear hormone receptor as a common link between genes that are regulated by HSF-1 in a HS-dependent manner, and an epidermal growth factor receptor as a common link between genes that are regulated by HSF-1 in a HS-independent manner. HSF-1 therefore coordinates various physiological processes in *C. elegans*, and HSF-1 activity may be coordinated across tissues by nuclear hormone receptor and epidermal growth factor receptor signaling.

**Conclusion:**

This work provides genome-wide HSF-1 regulatory networks in *C. elegans* that are both heat stress-dependent and -independent. We show that HSF-1 is responsible for regulating many genes outside of classical heat stress-responsive genes, including genes involved in development, metabolism, and aging. The findings that a nuclear hormone receptor may coordinate the HS-induced HSF-1 transcriptional response, while an epidermal growth factor receptor may coordinate the HS-independent response, indicate that these factors could promote cell non-autonomous signaling that occurs through HSF-1. Finally, this work highlights the genes involved in cuticle structure as important HSF-1 targets that may play roles in promoting both cytoprotection as well as longevity.

**Electronic supplementary material:**

The online version of this article (doi:10.1186/s12864-016-2837-5) contains supplementary material, which is available to authorized users.

## Background

When organisms are exposed to protein-denaturing stressors such as heat, the heat shock response (HSR) is engaged to manage protein damage and restore proteostasis [1]. The HSR is highly conserved across species and is regulated by the transcription factor heat shock factor 1 (HSF1). During basal conditions, HSF1 exists as a monomer in the cytoplasm and nucleus, and during stress conditions undergoes trimerization and accumulation in the nucleus, where it binds to heat shock elements in the promoters of heat shock protein (*hsp)* genes [2]. HSPs primarily act as molecular chaperones which refold the misfolded proteins that accumulate during stress, but they can also have essential functions in protein synthesis, processing, and degradation [3, 4]. Thus the HSR, and HSPs, play a large role in maintaining organismal proteostasis.

The soil-dwelling, free-living, nematode *Caenorhabditis elegans* is a powerful model organism that has provided insights into the regulation of a number of stress response pathways, including the HSR. HSF-1, the *C. elegans* homolog to mammalian HSF1, contains conserved N-terminal DNA-binding and trimerization domains, as well as a putative transactivation domain at the C-terminus [5]. It has recently been shown that the same activity steps required for mammalian HSF1 activation, including trimerization, hyperphosphorylation, and induction of DNA-binding, are also required for worm HSF-1 activation [6, 7].

Studies in *C. elegans* show that HSF-1 plays a central role not only in the HSR, but also in contributing to organismal physiology. HSF-1 is essential to worm viability, as a truncated mutant that lacks the C-terminal putative activation domain is defective in chaperone induction and egg laying, and also has a decreased lifespan [5]. In addition, this strain has a temperature-sensitive developmental arrest phenotype, with arrest occurring at the L2-L3 transition [5]. Various experiments using *hsf-1* RNA interference (RNAi) have shown that HSF-1 regulates the expression of specific *hsp* genes upon heat shock (HS), and have also implicated a non-stress-induced role for HSF-1 in processes including development, metabolism, and longevity [5, 8–14]. Interestingly, studies in *C. elegans* have identified the HSR as a cell non-autonomous process that requires thermosensory neurons for *hsp* induction [15]. Upon the completion of sequencing of the *C. elegans* genome, over 40 % of the predicted protein products were found to be significantly conserved in other organisms [16], and many signaling pathways are conserved [17]. *C. elegans* is thus an excellent model system for studying the role of HSF-1 in stress responses and other physiological processes in a simple multicellular organism.

In this study, we have performed RNA-sequencing (RNA-seq) with synchronous larval stage L4 wild-type *C. elegans* fed empty vector (EV) control RNAi or *hsf-1* RNAi treated with or without HS. We show that significant numbers of genes are upregulated as well as downregulated by HSF-1 under both conditions. In addition to *hsp* genes, HSF-1 is required for the regulation of genes involved in a wide variety of cellular processes including cytoprotection, development, metabolism, and aging. Network analysis points to possible routes by which HSF-1 signaling may be coordinated across tissues. A nuclear hormone receptor may coordinate the HS-induced HSF-1 transcriptional response, while an epidermal growth factor receptor may coordinate the HS-independent response. Surprisingly, the top HSF-1-regulated gene category, both with and without heat stress, is cuticle structure. This result, together with other recent studies, thus links regulation of the extracellular matrix to HSF-1, cytoprotection, and longevity.

## Results

### Experimental set-up for genome-wide analysis of regulation of gene expression by HSF-1

Previous experiments have shown that HSF-1 regulates the expression of specific *hsp* genes upon HS, and have also implicated a non-stress-induced role for HSF-1 in development, metabolism, and longevity [5, 8–14]. To examine HS-dependent vs. -independent gene regulation by HSF-1 on a genome-wide level, we used whole transcriptome RNA-sequencing. We treated synchronous L1 larval stage nematodes with RNAi against *hsf-1* [indicated as *hsf-1*(-)] or with an EV control plasmid [indicated as *hsf-1*(+)] until the L4 larval stage. At the L4 stage, we then treated nematodes from both groups with or without a 30 min 33 °C HS, as diagrammed (see Additional file 1: Figure S1a). Experiments were performed in biological duplicates. The L4 stage was chosen for our studies as this is a time when the response to HS is strong, prior to a sharp decline that occurs shortly after the transition to adulthood [18, 19]. These treatment conditions, optimized for our studies, resulted in a ~9- log_2_-fold induction of the *hsp-70* gene *C12C8.1*, a classical HSF-1 target gene, in *hsf-1*(+) animals treated with HS (see Additional file 1: Figure S1b, black bars). As expected, RNAi against *hsf-1* blunted *hsp-70* induction by HS (see Additional file 1: Figure S1b, purple bars). The efficiency of our RNAi feeding strategy was assessed by testing the effects of *hsf-1* RNAi on transcription driven by two heat shock protein promoter- GFP reporter worm strains (see Additional file 1: Figure S1c-d). HS increases GFP expression, and this effect is dependent on HSF-1 as demonstrated with *hsf-1* RNAi. Using an HSF-1::GFP overexpression worm strain, we also show that HSF-1 protein levels are reduced 80 % in response to *hsf-1* RNAi treatment (see Additional file 1: Figure S1e-f). Overall, these data validate our HS treatment conditions and RNAi feeding strategy.

Cluster analysis performed on biological replicate RNA-seq samples revealed conserved patterns of expression induced by each treatment condition (see Additional file 1: Figure S2). We normalized each condition to the *hsf-1*(+);-HS control in order to determine fold changes in relative RNA abundance (see Additional file 1: Figure S3). A complete list of the significant genes altered in response to each condition, after normalization to the control, is also provided (see Additional file 2: Table S1). Volcano plot analyses show that while a limited group of genes for each comparison have a log_2_-fold change of 6 or higher, the majority of genes have a log_2_ -fold change of approximately 4 or less (see Additional file 1: Figure S4a-c). As growth temperature and HSF-1 expression levels can affect the rate of development in the worm [20], we verified that our observed gene expression changes were not simply due to a change in the rate of development between each treatment condition. To do this, we analyzed several genes that are known to be differentially expressed during development and molting, including *abu-11*, *wrt-2*, *his-24*, *lin-29*, *abu-10*, *abu6*, *pqn-47*, *pin-42*, *ptr-3*, *abu-8*, *abu-7*, and *sdz-37* [21], and detected no significant expression differences in these genes across treatment groups (see Additional file 1: Figure S5). Overall, our data indicate that the worms in our four treatment conditions are developmentally synchronous to one another, and that the biological replicates for each condition share a similar expression profile, thus validating our experimental conditions.

Next, to visualize total HS-dependent vs. -independent gene expression regulated by HSF-1, we constructed a Venn diagram with the differentially expressed genes for each condition which were determined to be statistically significant as compared to the *hsf-1*(+);-HS control (see Additional file 1: Figure S6). The shaded areas of the Venn diagram correspond to HS-dependent and -independent processes regulated by HSF-1 (as indicated by the red and pink shaded areas in Figure S6, respectively), and these are the transcripts that we have focused our subsequent analyses on. Altogether, we found that 942 genes are significantly regulated by HSF-1 during HS (Figure S6, red shaded area), and that 2,436 genes are significantly regulated by HSF-1 independently of HS (Figure S6, pink shaded area), highlighting that HSF-1 regulates both HS-dependent and -independent transcriptional processes. Interestingly, only 4 genes are significantly regulated by HS independently of HSF-1. HSF-1 is thus not only an essential transcriptional regulator for a majority of the genes altered by HS, but global RNA expression analysis supports a gene-regulatory role for HSF-1 under both heat stress and non-stress conditions. A greater number of gene changes that depend on HSF-1 are independent of heat stress, thus highlighting the important role of HSF-1 in regulating gene expression under non-stress conditions.

### Genes that are regulated by HSF-1 in response to HS

#### Genes that are normally upregulated by HSF-1 in response to HS

**Fig. 1:**
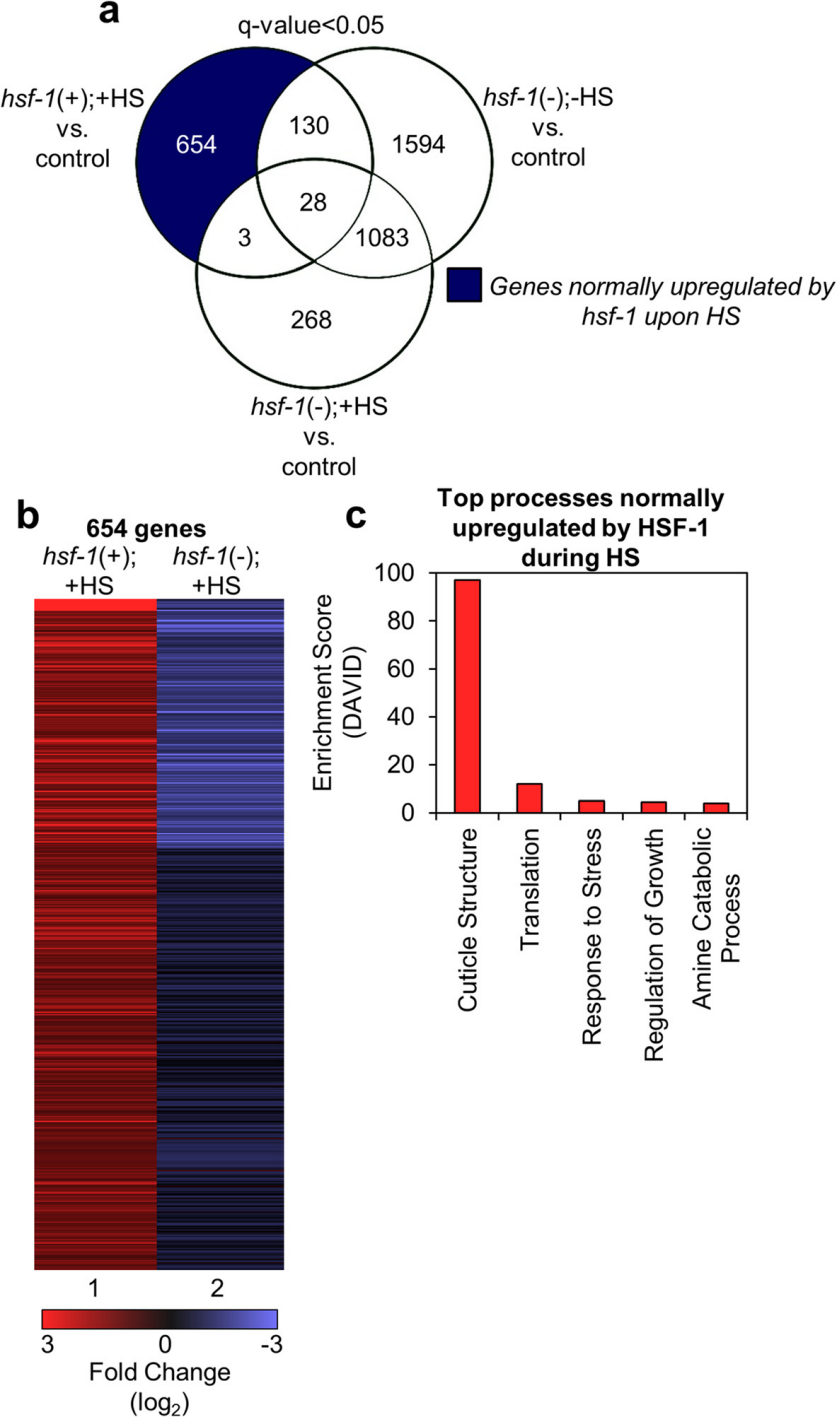
Genes that are normally upregulated by HSF-1 in response to HS. **a** The Venn diagram shows the overlap among genes that were found to be significantly upregulated (q-value < 0.05) for each of the indicated comparisons between samples. The dark blue shaded area includes genes that are normally upregulated by HSF-1 upon HS. The q-value is the FDR-adjusted *p*-value of the test statistic, as determined by the Benjamini-Hochberg correction for multiple testing. **b** Hierarchical clustering of the genes normally upregulated by HSF-1 upon HS*.* Lane 1 corresponds to the fold change of the 654 genes found in the dark blue section of the Venn diagram in (**a**) in the *hsf-1*(+);+HS vs. control samples. As a comparison, lane 2 corresponds to the fold change of the same genes found in lane 1, but in the *hsf-1*(-);+HS vs. control samples, as determined by RNA-seq. The heat map was organized using Cluster 3 by k-means and Euclidean distance. **c** Top processes normally upregulated by HSF-1 during HS. The genes found in the dark blue section of the Venn diagram in (**a**) were classified by Gene Ontology terms that were determined using DAVID

To determine whether HSF-1 can affect gene expression in both a positive and negative fashion, we separated out the positively vs. negatively regulated genes and analyzed them by Venn diagram. We find that 654 transcripts are normally upregulated by HSF-1 upon HS (Fig. 1a, dark blue). We next examined these 654 genes in more depth. The top 15 genes in this category are listed in Table 1 (a complete list of the 654 significantly upregulated genes is provided, see Additional file 3: Table S2). Included in the top 15 upregulated transcripts are 3 *hsp-70* family genes and 6 *hsp-16* family genes, all with log_2_-fold changes greater than 6. The presence of chaperone genes in our top 15 hits was expected, and gave us confidence in our experimental strategy. Aside from chaperone genes, there are a number of non-chaperones included in the top 15 most upregulated genes, including the nucleosome remodeling factor complex member *nurf-1* [22]*,* the predicted collagen gene *col-149*, and various genes of unknown function (Table 1). Therefore, the top 15 genes regulated by HSF-1 under HS conditions include 9 *hsp* genes and 6 genes with diverse functions.

**Table 1:** Top 15 genes normally upregulated by HSF-1 in response to HS

	Transcript ID	Gene Name	Fold Change (log_2_) *hsf-1*(+);+HS vs. control	Description (WormBase)
1	*F44E5.5*	*F44E5.5*	8.06	*F44E5.5* encodes a member of the Hsp70 family of heat shock proteins
2	*F44E5.4*	*F44E5.4*	7.87	*F44E5.4* encodes a member of the Hsp70 family of heat shock proteins
3	*Y46H3A.3*	*hsp-16.2*	7.53	*hsp-16.2* encodes a 16-kD heat shock protein (HSP) that is a member of the hsp16/hsp20/alphaB-crystallin (HSP16) family of heat shock proteins
4	*Y46H3A.2*	*hsp-16.41*	7.41	*hsp-16.41* encodes a 16-kD heat shock protein (HSP) that is a member of the hsp16/hsp20/alphaB-crystallin (HSP16) family of heat shock proteins
5	*T27E4.2*	*hsp-16.11*	7.31	*hsp-16.11* encodes a 16-kD heat shock protein (HSP) that is a member of the hsp16/hsp20/alphaB-crystallin (HSP16) family of heat shock proteins
6	*C12C8.1*	*hsp-70*	7.15	*hsp-70* encodes a heat-shock protein that is a member of the HSP70 family of molecular chaperones
7	*T27E4.8*	*hsp-16.1*	6.9	*hsp-16.1* encodes a 16-kD heat shock protein (HSP) that is a member of the hsp16/hsp20/alphaB-crystallin (HSP16) family of heat shock proteins
8	*T27E4.3*	*hsp-16.48*	6.77	*hsp-16.48* encodes a 16-kD heat shock protein (HSP) that is a member of the hsp16/hsp20/alphaB-crystallin (HSP16) family of heat shock proteins
9	*Y38E10A.13*	*nspe-1*	6.54	*nspe-1* is a nematode-specific peptide that has an unknown function
10	*T27E4.9*	*hsp-16.49*	6.5	*hsp-16.49* encodes a 16-kD heat shock protein (HSP) that is a member of the hsp16/hsp20/alphaB-crystallin (HSP16) family of heat shock proteins
11	*F26H11.2*	*nurf-1*	4.15	*nurf-1* encodes the *C. elegans* ortholog of *Drosophila* NURF301, a component of the NURF chromatin remodeling complex
12	*ZC21.10*	*ZC21.10*	3.84	Unknown function
13	*D2013.8*	*scp-1*	3.68	*scp-1* encodes PTC-related protein that contains a sterol-sensing domain related to human Sterol regulatory element binding protein (SREBP) cleavage activating protein
14	*R107.5*	*R107.5*	3.29	Unknown function
15	*B0024.1*	*col-149*	3.01	*col-149* is predicted to be a structural constituent of the cuticle

The transcript ID, gene name, fold change as compared to the *hsf-1*(+);-HS control, and description as provided from WormBase, are listed for the 15 genes with the highest positive fold changes in expression. A complete list of significantly upregulated transcripts is given (see Additional file 2: Table S1)

We then further examined the induction characteristics of the top 15 HSF-1-dependent genes induced by HS. The log_2_-fold changes for a subset of these genes are plotted (see Additional file 1: Figure S7a, black bars) and compared to the expression of the same genes in the presence of HS but in the absence of *hsf-1* (see Additional file 1: Figure S7a, purple bars). The fact that *hsf-1* RNAi completely eliminates HS-inducibility of these genes highlights their dependency on HSF-1. Independent quantitative RT-PCR (qRT-PCR) for the same subset of highly induced genes (see Additional file 1: Figure S7b) validates our RNA-seq data.

In order to better visualize the global patterns of transcript upregulation by HSF-1 in response to HS, we constructed a heat map of the 654 significantly upregulated genes (Fig. 1b, lane 1). Interestingly, and consistent with the data for a set of the top 15 upregulated genes (see Additional file 1: Figure S7a-b), genes that are normally upregulated by HSF-1 upon HS are either unchanged or downregulated under HS conditions upon *hsf-1* knockdown on a global level (Fig. 1b, lane 2). The fact that many of the 654 HS-induced genes that require HSF-1 are downregulated upon *hsf-1* knockdown implies that HSF-1 may play a role in the basal regulation of these genes, which is then enhanced upon HS. Together, these data demonstrate the HSF-1 dependency of most HS-induced genes.

To identify the various functional processes normally upregulated by HSF-1 during HS, we used the Database for Annotation, Visualization, and Integrated Discovery (DAVID) classification tool to define the top 5 gene ontology terms for all of the 654 genes found to be significantly upregulated (Fig. 1c). The complete output from DAVID is also provided (see Additional file 4: Table S3). Surprisingly, the top functional category, with an enrichment score of 97, contains genes involved in cuticle structure. The next four categories, all with enrichment scores under 12, include genes involved in translation, the response to stress, the regulation of growth, and amine catabolic processes. We thus find that the largest functional category of genes regulated by HS is not the expected heat stress-responsive gene-set, but instead genes associated with forming cuticle structure.

We next tested the effects of a cuticle collagen gene, *col-123*, on induction of the HSR by measuring *hsp-70* promoter activity in p*hsp-70*::GFP worms (see Additional file 1: Figure S8a-c). We see that *col-123* RNAi decreases HS-induced *hsp-70* promoter activity, and may control tissue-specific regulation of the HSR. Testing the effects of other collagen genes on regulation of the HSR may provide insight into a signaling role for collagens in coordinating stress responses.

#### Genes that are normally downregulated by HSF-1 in response to HS

**Fig. 2:**
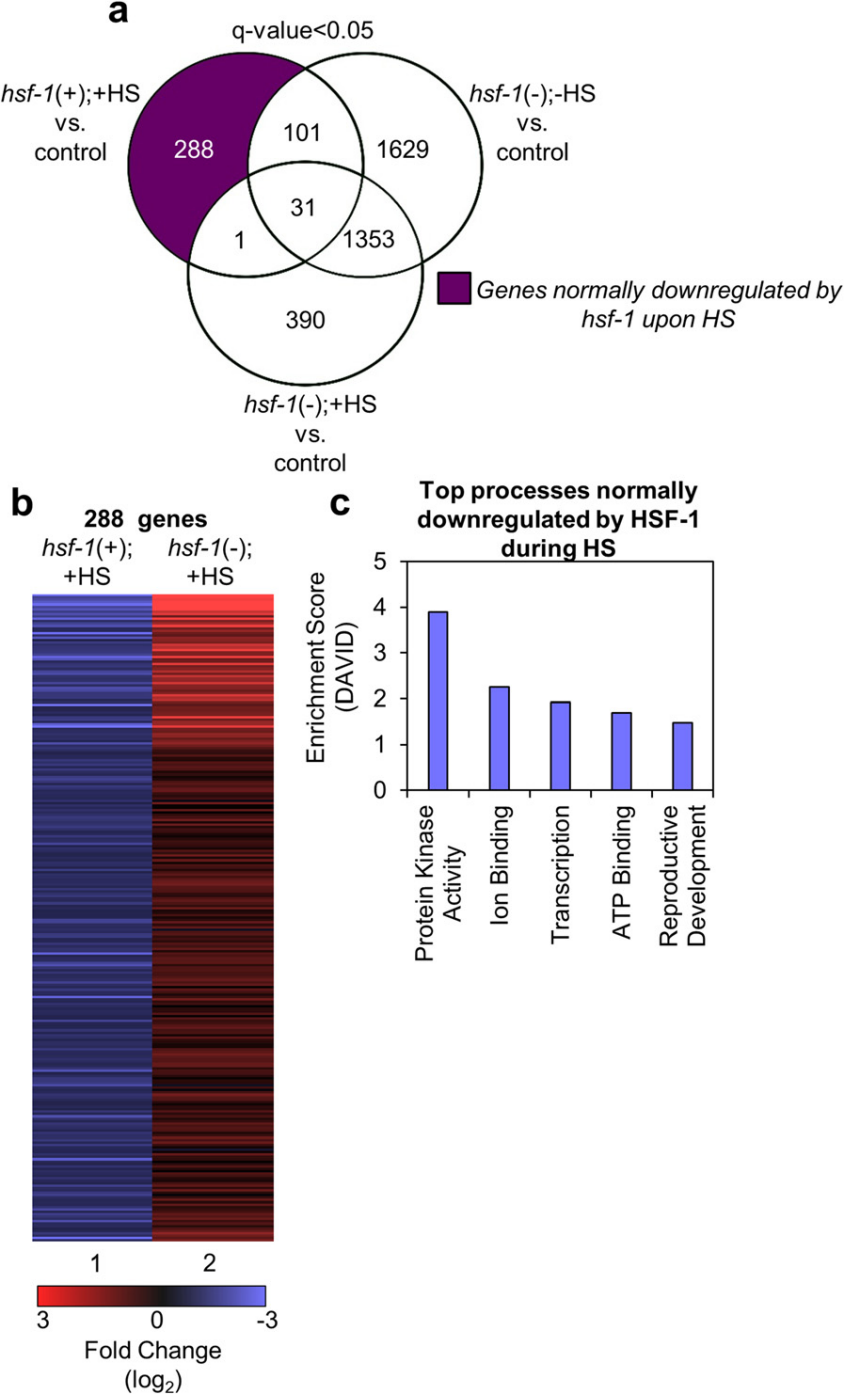
Genes that are normally downregulated by HSF-1 in response to HS. **a** The Venn diagram shows the overlap among genes that were found to be significantly downregulated (q-value < 0.05) for each of the indicated comparisons between samples. The dark purple shaded area includes genes that are normally downregulated by HSF-1 upon HS. The q-value is the FDR-adjusted *p*-value of the test statistic, as determined by the Benjamini-Hochberg correction for multiple testing. **b** Hierarchical clustering of the genes normally downregulated by HSF-1 upon HS*.* Lane 1 corresponds to the fold change of the 288 genes found in the dark purple section of the Venn diagram in (**a**) in the *hsf-1*(+);+HS vs. control samples. As a comparison, lane 2 corresponds to the fold change of the same genes found in lane 1, but in the *hsf-1*(-);+HS vs control samples, as determined by RNA-seq. The heat map was organized using Cluster 3 by k-means and Euclidean distance. **c** Top processes normally downregulated by HSF-1 during HS. The genes found in the dark purple section of the Venn diagram in (**a**) were classified by Gene Ontology terms that were determined using DAVID

We next examined the genes separated out from the Venn diagram analysis to be normally downregulated by HSF-1 upon HS (Fig. 2a). We find that there are 288 transcripts in this group (Fig. 2a, dark purple). The top 15 genes normally downregulated by HSF-1 upon HS are listed in Table 2 (a complete list of the 288 significantly downregulated genes is provided, see Additional file 3: Table S2). There are a variety of distinct transcripts downregulated by HSF-1 during HS. The gene with the largest log_2_-fold decrease (-3.71) is *acs-2,* which encodes an acyl-CoA synthetase. This enzyme participates in breakdown of fatty acids into acyl-CoA in the mitochondria to allow for β-oxidation, thus increasing fat consumption [23]. Another downregulated gene is *dct-1,* which encodes a protein that has pro-apoptotic activity [24]. The tetraspanin family member *tsp-1* is also downregulated. The tetraspanin family of proteins is required for epithelial integrity in the worm and regulates cuticle formation [25]. Other downregulated genes include *fbxa-66* and *fbxa-21,* which encode FboxA proteins with unknown functions; *nep-26,* which encodes a zinc metallopeptidase that negatively regulates signaling peptides [26]; *glc-1,* which encodes a subunit of a glutamate-gated chloride channel [27]; and *delm-2,* which encodes an ortholog of an acid-sensing ion channel family member [28]. There are also multiple transcripts of unknown function in this gene group. Therefore, the top 15 genes normally downregulated by HSF-1 under HS conditions have a diverse set of functions.

**Table 2:** Top 15 genes normally downregulated by HSF-1 in response to HS

	Transcript ID	Gene Name	Fold Change (log_2_) *hsf-1*(+);+HS vs. control	Description (WormBase)
1	*F28F8.2*	*acs-2*	−3.71	*acs-2* encodes an acyl-CoA synthetase; by homology, ACS-2 is predicted to catalyze conversion of a fatty acid to Acyl-CoA for subsequent beta oxidation
2	*W09G12.7*	*W09G12.7*	−3.28	Unknown function
3	*C07A4.2*	*C07A4.2*	−2.85	Unknown function
4	*C14F5.1*	*dct-1*	−2.84	*dct-1* encodes a protein with similarity to the mammalian BNIP3 proteins that interact with Bcl-2 and the Adenovirus E1B proteins which have been shown to have pro-apoptotic activity
5	*C02F5.8*	*tsp-1*	−2.81	*tsp-1* is a part of the tetraspanin family that encodes an ortholog of a human CD151 molecule
6	*Y54F10BM.11*	*fbxa-66*	−2.63	*fbxa-66* encodes an FboxA protein that has an unknown function
7	*Y119D3B.9*	*fbxa-21*	−2.55	*fbxa-21* encodes an FboxA protein that has an unknown function
8	*K08D9.4*	*K08D9.4*	−2.54	Unknown function
9	*C49G7.7*	*C49G7.7*	−2.52	Unknown function
10	*T12A7.6*	*T12A7.6*	−2.42	Unknown function
11	*F33H12.7*	*F33H12.7*	−2.30	Unknown function
12	*Y47H10A.5*	*Y47H10A.5*	−2.14	Unknown function
13	*ZK970.1*	*nep-26*	−2.12	*nep-26* is a thermolysin-like zinc metallopeptidase found on the surface of cells that negatively regulates small signaling peptides
14	*F11A5.10*	*glc-1*	−2.07	*glc-1* is the alpha subunit of a glutamate-gated chloride channel
15	*C24G7.1*	*delm-2*	−2.06	*delm-2* encodes an ortholog of human acid-sensing ion channel family member 4, and is predicted to have sodium channel activity

The transcript ID, gene name, fold change as compared to the *hsf-1*(+);-HS control, and description as provided from WormBase are listed for the 15 genes with the highest negative fold changes in expression. A complete list of significantly downregulated transcripts is given (see Additional file 2: Table S1)

We then further studied the top 15 genes that are normally downregulated by HSF-1 upon HS. The log_2_-fold changes of a subset of the top 15 HSF-1-dependent genes repressed by HS are plotted (see Additional file 1: Figure S9a, black bars), and these data are compared to the expression of the same genes in the presence of HS but in the absence of *hsf-1* (see Additional file 1: Figure S9a, purple bars). Interestingly, we found that all of the genes that are downregulated by HS in the presence of HSF-1 are upregulated by HS in the absence of HSF-1. One way this could occur is if HSF-1 normally suppresses the expression of these genes, and HS-activated HSF-1 suppresses them even further. To verify our RNA-seq data, we performed independent qRT-PCR for the same subset of highly downregulated genes, and found that the qRT-PCR data was consistent with our RNA-seq data (see Additional file 1: Figure S9b).

To visualize the patterns of transcripts that are normally downregulated by HSF-1 upon HS, we constructed a heat map to visualize the log_2_-fold changes of the 288 significantly downregulated genes (Fig. 2b, lane 1). As a comparison, the expression of the same transcripts under HS conditions upon *hsf-1* knockdown is shown (Fig. 2b, lane 2). As with the data for the top downregulated genes (see Additional file 1: Figure S9a-b), many of the 288 genes that are normally downregulated by HSF-1 under HS conditions are conversely upregulated by HS in the absence of HSF-1. Thus, HS can have completely opposite effects on gene expression depending on the presence or absence of HSF-1.

Upon DAVID analysis of gene ontology terms for the repressed genes, the top 5 functional categories all had enrichment scores of 4 or lower. These functional categories include genes that encode proteins with kinase activity, ion binding activity, transcription, ATP-binding activity, and reproductive development (Fig. 2c). The complete output from DAVID is also provided (see Additional file 4: Table S3). Overall, these results show that a diverse group of genes are normally suppressed by HSF-1 during HS.

### Genes that are regulated by HSF-1 independently of HS

#### Genes that are normally upregulated by HSF-1 independently of HS

**Fig. 3:**
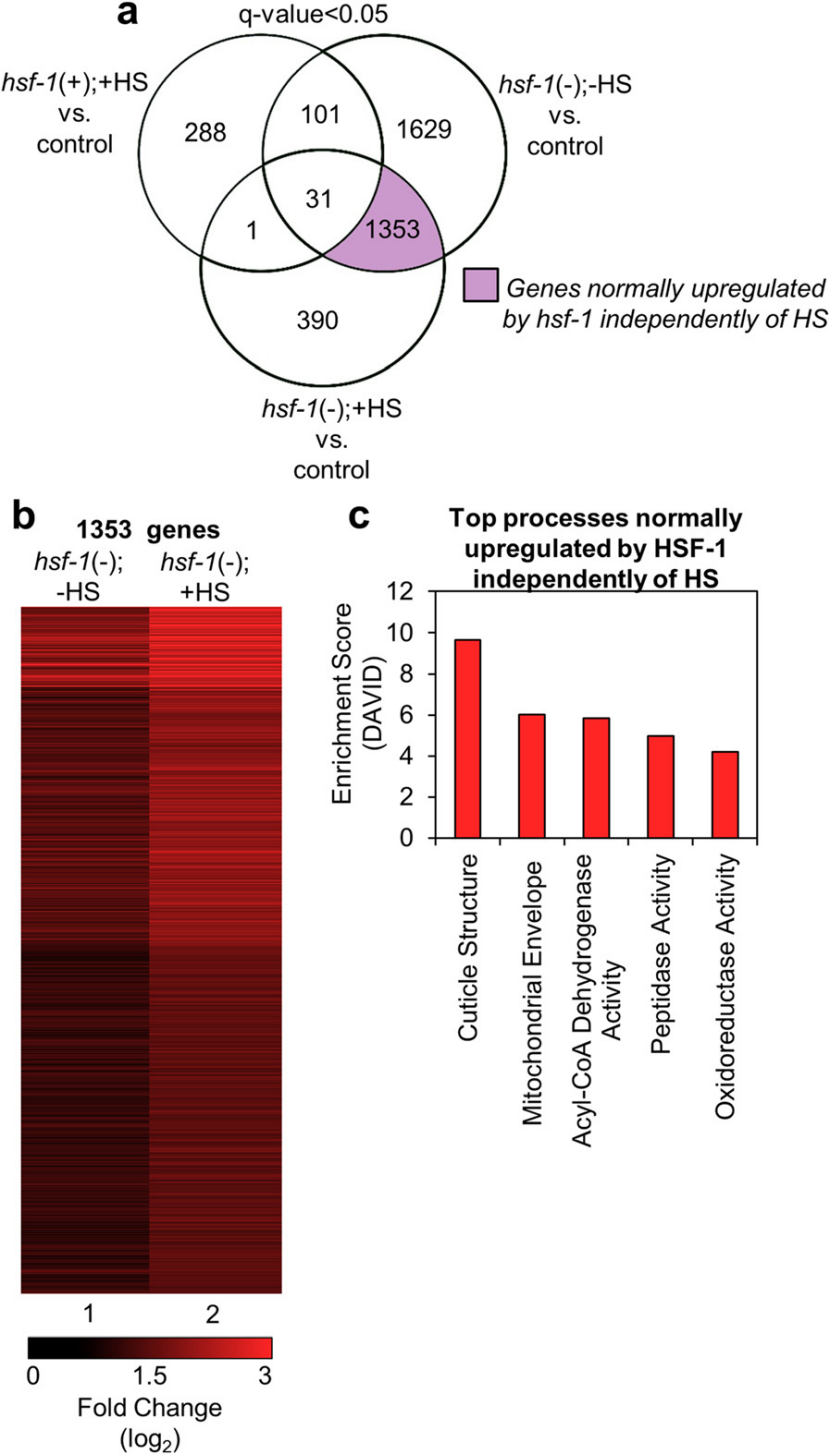
Genes that are normally upregulated by HSF-1 independently of HS. **a** The Venn diagram shows the overlap among genes that were found to be significantly downregulated (q-value < 0.05) for each of the indicated comparisons between samples. The light purple shaded area includes genes that are downregulated upon treatment with *hsf-1* RNAi, and are likely normally induced by HSF-1 independently of heat shock, therefore are referred to as upregulated genes. The q-value is the FDR-adjusted *p*-value of the test statistic, as determined by the Benjamini-Hochberg correction for multiple testing. **b** Hierarchical clustering of the genes normally upregulated by HSF-1 independently of HS. The fold change of genes found in the light purple section of the Venn diagram in (**a**) were determined by RNA-seq to be downregulated in response to *hsf-1* RNAi, and would thus normally be upregulated by HSF-1. Lane 1 corresponds to the fold change of these genes in the control vs. *hsf-1*(-);-HS samples, and as a comparison, lane 2 corresponds to each the fold change of the same genes found in lane 1 but in the control vs. *hsf-1*(-);+HS samples. The heat map was organized using Cluster 3 by k-means and Euclidean distance. **c** Top processes normally upregulated by HSF-1 independently of HS. The genes found in the light purple section of the Venn diagram were classified by Gene Ontology terms that were determined using DAVID

While HSF-1 has been historically studied for its role in regulating responses elicited by HS, HSF-1 also has functions that are independent of HS including roles in development, metabolism, and longevity [7, 9–11]. To identify processes upregulated by HSF-1 independently of HS, we examined the 1,353 genes from the Venn diagram that we determined to be downregulated in response to *hsf-1* RNAi independently of HS, suggesting that they are normally upregulated by HSF-1 (Fig. 3a, light purple). In order to gain insight into the normal HSF-1-regulatory role of these HS-independent genes, we reversed our data comparison [control vs. *hsf-1*(-);-HS] to obtain the fold change, as this gene group is shown to be downregulated in response to *hsf-1* RNAi and would thus normally be upregulated by HSF-1.

The top 15 genes that are normally upregulated by HSF-1 under non-stress conditions are listed in Table 3 (a complete list of the significantly upregulated genes is provided, see Additional file 3: Table S2). Surprisingly, a group of vitellogenin lipid transporter transcripts (*vit*-1, -3,-4, and -5) are among the top 15 genes. Vitellogenins are made in the intestine of late larval/early adult hermaphrodites and are taken up by the germ cells to provide nourishment to embryos [29]. Other top upregulated genes include *acdh-1,* which encodes a short chain acyl-CoA dehydrogenase and may play a role in energy production [30, 31]; *K11G9.3,* which is predicted to be an ortholog of human butyrylcholinesterase; *folt-2,* which encodes a folate transporter [32]; *ilys-5,* which is predicted to have lysozyme activity; *ZC266.1,* which is predicted to have G-protein coupled receptor activity; *fat-7*, which encodes a fatty acid desaturase [33]; *K10B2.2,* which is predicted to have carboxypeptidase activity; *Y52E8A.4*, which encodes the ortholog to a major facilitator superfamily; *ugt-22,* which encodes the ortholog of a polypeptide predicted to have transferase activity; and two genes with unknown function. Therefore, a diverse set of genes, including vitellogenins and others, are normally upregulated by HSF-1 independently of HS.

**Table 3:** Top 15 genes normally upregulated by HSF-1 independently of HS

	Transcript ID	Gene Name	Fold Change (log_2_) control vs. *hsf-1*(-);-HS	Description (WormBase)
1	*C55B7.4*	*acdh-1*	4.62	*acdh-1* encodes a short-chain acyl-CoA dehydrogenase. ACDH-1 is predicted to be a mitochondrial enzyme that catalyzes the first step of fatty acid beta-oxidation, and thus plays a key role in energy production
2	*F59D8.1*	*vit-3*	3.52	*vit-3* encodes a vitellogenin, a precursor of the lipid-binding protein related to vertebrate vitellogenins and mammalian ApoB-100, a core LDL particle constituent
3	*F59D8.2*	*vit-4*	3.47	*vit-4* is involved in embryo development and is predicted to have lipid transporter activity
4	*C04F6.1*	*vit-5*	3.39	*vit-5* encodes a vitellogenin, a lipid-binding protein precursor related to vertebrate vitellogenins and mammalian ApoB-100, a core LDL particle constituent
5	*K09F5.2*	*vit-1*	3.38	*vit-1* is predicted to have lipid transporter activity
6	*Y40H7A.10*	*Y40H7A.10*	3.37	Unknown function
7	*K11G9.3*	*K11G9.3*	3.15	*K11G9.3* encodes an ortholog of human butyrylcholinesterase
8	*F37B4.7*	*folt-2*	3.06	*folt-2* encodes a putative folate transporter and is orthologous to the human folate transporters SLC19A1, SLC19A2, and SLC19A3
9	*F22A3.6*	*ilys-5*	2.99	*ilys-5* is involved in embryo development and is predicted to have lysozyme activity
10	*ZC266.1*	*ZC266.1*	2.76	*ZC266.1* is predicted to have G-protein coupled receptor activity
11	*F10D2.9*	*fat-7*	2.64	*fat-7* encodes an essential delta-9 fatty acid desaturase that is required for the synthesis of monounsaturated fatty acids
12	*K10B2.2*	*K10B2.2*	2.55	*K10B2.2* encodes an ortholog of human cathepsin A and is predicted to have carboxypeptidase activity
13	*Y52E8A.4*	*Y52E8A.4*	2.46	*Y52E8A.4* encodes an ortholog of human major facilitator superfamily domain containing 11
14	*C08F11.8*	*ugt-22*	2.44	*ugt-22* encodes an ortholog of human UDP glucuronosyltransferase 1 family polypeptide, and is predicted to have transferase activity
15	*F54F7.2*	*F54F7.2*	2.44	Unknown function

The transcript ID, gene name, and fold change in gene expression in the comparison of the control vs*. hsf-*1(-);-HS are listed for the top 15 genes normally upregulated by HSF-1 independently of HS. Gene descriptions, as provided from WormBase, are also listed. A complete list of the significantly altered transcripts is given (see Additional file 2: Table S1)

We then further characterized the induction characteristics of the top 15 genes that are normally upregulated by HSF-1 independently of heat stress. The log_2_-fold changes of a subset of these top 15 genes are plotted (see Additional file 1: Figure S10a, orange bars), and are compared to the expression of the same genes in the presence of HS and absence of *hsf-1* (see Additional file 1: Figure S10a, purple bars). This data shows that HS does not affect the expression of these genes in the absence of *hsf-1*. The expression of these mRNAs was also verified with qRT-PCR, and the results are consistent with the RNA-seq data (see Additional file 1: Figure S10b).

To investigate a HS-independent role for HSF-1 in the induction of gene expression on a global level, we generated a heat map of all 1,353 genes found via Venn diagram to be normally upregulated by HSF-1 in the absence of HS (Fig. 3b, lane 1). As a comparison, genes that are normally upregulated by HSF-1 in the presence of HS are plotted (Fig. 3b, lane 2). The transcripts in this group that are normally upregulated by HSF-1 in the absence of HS remain upregulated or unchanged in the presence of HS, verifying the heat stress-independent induction of this gene group.

We next used DAVID to determine the top 5 functional processes that are normally upregulated by HSF-1 independently of HS. We found that cuticle structure was again the gene category with the highest enrichment score (9.7), as was also the case for the genes that are upregulated by HSF-1 during HS (Fig. 3c). This indicates that HSF-1 may regulate the basal expression of genes involved in cuticle structure, and that these genes are then further induced upon HS. Other functional processes, with enrichment scores less than 6, include genes that encode proteins involved in the mitochondrial envelope, acyl-CoA dehydrogenase activity, peptidase activity, and oxidoreductase activity. The complete output from DAVID is also available (see Additional file 5: Table S4). The mitochondrial envelope, acyl-CoA dehydrogenase activity, peptidase activity, and oxidoreductase activity are all processes that can be linked to metabolism, further substantiating a functional role for HSF-1 in regulating this process.

#### Genes that are normally downregulated by HSF-1 independently of HS

**Fig. 4:**
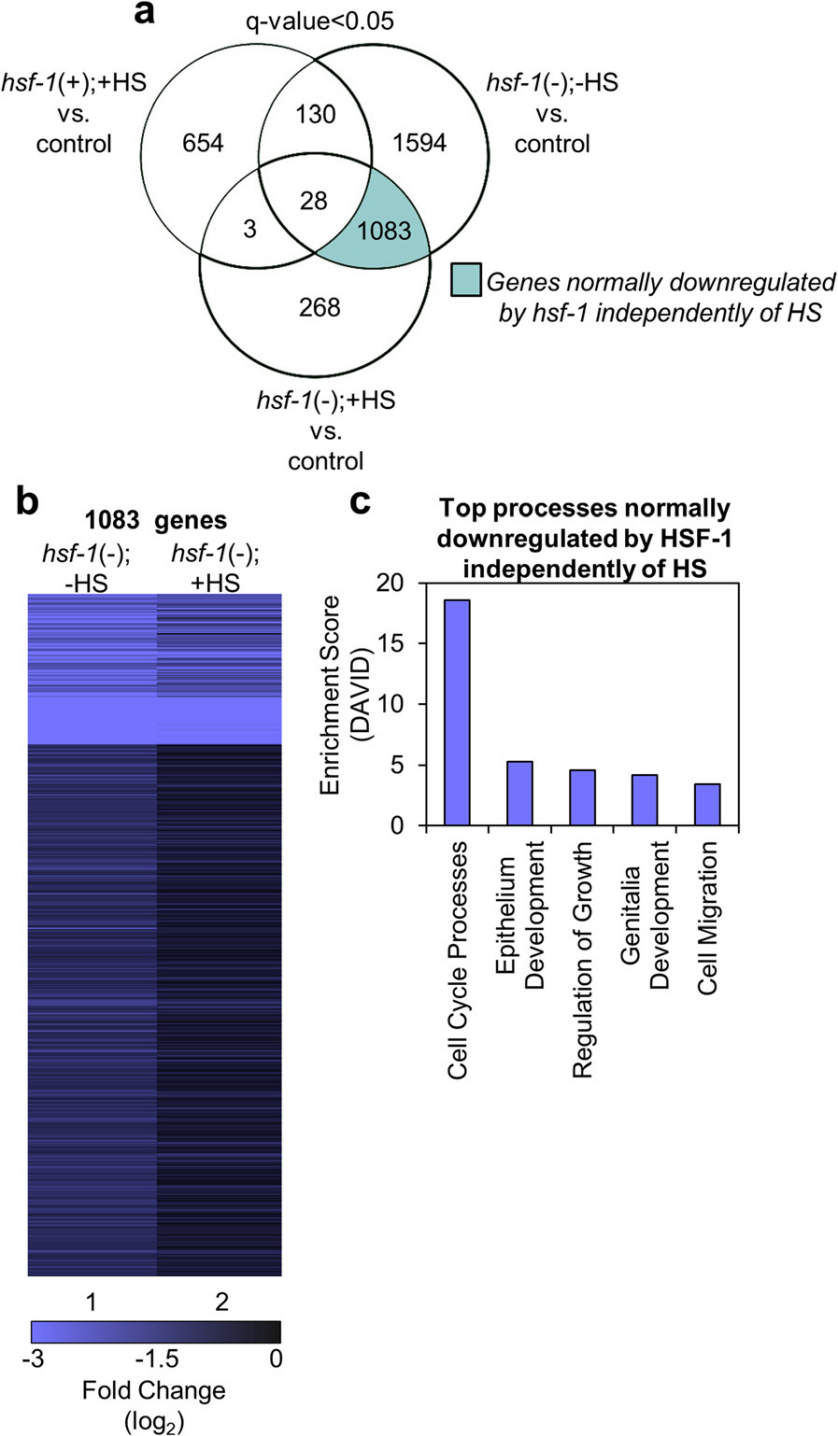
Genes that are normally downregulated by HSF-1 independently of HS. **a** The Venn diagram shows the overlap among genes that were found to be significantly upregulated (q-value < 0.05) for each of the indicated comparisons between samples. The light blue shaded area includes genes that are upregulated upon treatment with *hsf-1* RNAi, and are likely normally suppressed by HSF-1 independently of heat shock, therefore are referred to as downregulated genes. The q-value is the FDR-adjusted *p*-value of the test statistic, as determined by the Benjamini-Hochberg correction for multiple testing. **b** Hierarchical clustering comparing the genes normally suppressed by HSF-1 independently of HS*.* The fold change of genes found in the light blue section of the Venn diagram in (**a**) were determined by RNA-seq to be upregulated in response to *hsf-1* RNAi, and would thus normally be suppressed by HSF-1. Lane 1 corresponds to the fold change of these genes in the control vs. *hsf-1*(-);-HS samples, and as a comparison, lane 2 corresponds to each the fold change of the same genes found in lane 1 but in the control vs. *hsf-1*(-);+HS samples. The heat map was organized using Cluster 3 by k-means and Euclidean distance. **c** Top processes normally downregulated by HSF-1 independently of HS. The genes found in the light blue section of the Venn diagram were classified by Gene Ontology terms that were determined using DAVID

We next examined the 1,083 genes from the Venn diagram that we determined to be upregulated in response to *hsf-1* RNAi independently of HS, suggesting that they are normally downregulated by HSF-1 (Fig. 4a, light blue). We reversed our data comparison [control vs *hsf-1*(-);-HS] to obtain the fold change, as this gene group is shown to be upregulated in response to *hsf-1* RNAi and would thus normally be downregulated by HSF-1. The top 15 genes in this category are listed in Table 4 (a complete list of the significantly downregulated genes is provided, see Additional file 3: Table S2). There are a variety of transcript types in this list, including *T22F3.11,* which is an mRNA that encodes the ortholog of the human solute carrier family 17; *eol-1,* which regulates olfactory learning [34]; *BO348.2,* which encodes an ortholog of human lipopolysaccharide-induced TNF factor; *col-158,* which encodes a structural constituent of the cuticle; *fbxa-163* and *T08E11.1,* which encode proteins that contain F-box motifs predicted to be important for protein-protein interactions; *clec-174* and *clec-13*, which encode carbohydrate binding proteins; *srg-31,* which encodes a protein involved in embryo development; *clec-60,* which encodes a protein involved in the immune response; *B0507.8,* which encodes an ortholog of human cingulin-like 1; *clec-13,* which is predicted to have carbohydrate binding activity; *F22F12.1,* which encodes an ortholog of human GRB10 interacting GYF protein 2; and *Y47H10A.5* and *ZK355.8,* which both have unknown functions. Overall, we find that a diverse set of mRNAs are normally downregulated by HSF-1 independently of HS, indicating that HSF-1 may normally suppress a variety of cellular processes in a HS-independent manner.

**Table 4:** Top 15 genes normally downregulated by HSF-1 independently of HS

	Transcript ID	Gene Name	Fold Change (log_2_) control vs. *hsf-1*(-);-HS	Description (WormBase)
1	*T22F3.11*	*T22F3.11*	−5.85	*T22F3.11* encodes an ortholog of human solute carrier family 17
2	*T26F2.3*	*eol-1*	−5.38	*eol-1* is required in the URX sensory neurons for inhibition of olfactory learning
3	*B0348.2*	*B0348.2*	−5.32	*B0348.2* encodes an ortholog of human lipopolysaccharide-induced TNF factor
4	*Y47H10A.5*	*Y47H10A.5*	−5.17	Unknown function
5	*D2023.7*	*col-158*	−5.17	*col-158* is predicted to be a structural constituent of the cuticle
6	*C08E3.6*	*fbxa-163*	−4.95	*fbxa-163* encodes a protein containing an F-box, a motif predicted to mediate protein-protein interactions
7	*F07E5.9*	*F07E5.9*	−4.90	Unknown function
8	*Y46C8AL.2*	*clec-174*	−4.89	*clec-174* is predicted to have carbohydrate binding activity
9	*T07H8.5*	*srg-31*	−4.84	*srg-31* is involved in embryo development and is predicted to have transmembrane signaling receptor activity
10	*ZK666.6*	*clec-60*	−4.81	*clec-60* appears to play a role in the innate immune response to some bacterial pathogens
11	*B0507.8*	*B0507.8*	−4.64	*B0507.8* encodes an ortholog of human cingulin-like 1
12	*ZK355.8*	*ZK355.8*	−4.60	Unknown function
13	*H16D19.1*	*clec-13*	−4.50	*clec-13* is predicted to have carbohydrate binding activity
14	*T08E11.1*	*T08E11.1*	−4.37	*T08E11.1* encodes a protein containing an F-box motif predicted to mediate protein-protein interactions
15	*F22G12.1*	*F22G12.1*	−4.35	*F22G12.1* encodes an ortholog of human GRB10 interacting GYF protein 2

The transcript ID, gene name, and fold change in gene expression in the comparison of the control vs *hsf-1*(-);-HS are listed for the top 15 genes normally downregulated by HSF-1 independently of HS. Gene descriptions, as provided from WormBase, are also listed. A complete list of the significantly altered transcripts is given (see Additional file 2: Table S1)

The induction characteristics of the top 15 genes in this category were then analyzed. The log_2_-fold changes from the RNA-seq data for a subset these genes are plotted (see Additional file 1: Figure S10c, orange bars), and are compared to the expression of the same genes in the presence of HS in the absence of *hsf-1* (see Additional file 1: Figure S10c, purple bars). The expression of these mRNAs was also verified with qRT-PCR, and the results are consistent with the RNA-seq data (see Additional file 1: Figure S10d). Altogether, these data confirm that HS does not affect the expression of these genes in the absence of *hsf-1*.

To investigate a HS-independent role for HSF-1 in the suppression of gene expression, we constructed a heat map of all 1,083 genes found via Venn diagram to be normally suppressed by *hsf-1* in the absence of HS (Fig. 4b, lane 1). As a comparison, the expression patterns of the same transcripts suppressed by *hsf-1* in the presence of HS are shown (Fig. 4b, lane 2). The transcripts in this group that are significantly downregulated by HSF-1 in the absence of HS remain downregulated or unchanged by HSF-1 in the presence of HS, further verifying that regulation of this subset of genes by HSF-1 is independent of HS.

We next used DAVID to determine the functional processes that are normally downregulated by HSF-1 independently of HS. Genes involved in cell cycle processes were most abundant, with an enrichment score of 19, followed by genes involved in epithelium development, regulation of growth, genitalia development, and cell migration, all with enrichment scores under 6 (Fig. 4c). The complete output from DAVID is also available (see Additional file 5: Table S4). Cell cycle processes, epithelium development, regulation of growth, genitalia development, and cell migration are all processes that can be linked to development, thus confirming a HS-independent role for HSF-1 in development.

## Discussion

### Regulation of gene expression by HSF-1

*C. elegans* is a useful model organism for identifying regulatory processes that are shared between species. While the transcription factor HSF-1 has classically been studied as a factor that is responsive to cytoplasmic proteotoxic stress, it is becoming increasingly evident that this transcription factor also has major non-stress-induced roles in coordinating gene expression. With the RNA-sequencing experiments performed here, we confirm that HSF-1 can regulate gene expression under both heat stress and non-stress conditions. In addition to genes that are classically stress-responsive, such as chaperones, our work shows that HSF-1 also regulates sets of genes involved in a variety of cellular processes including metabolism, development, and longevity.

### Cuticle structure genes are normally upregulated by HSF-1 via HS-dependent and -independent mechanisms

A surprising result of our study is that genes controlling cuticle structure comprise the largest gene ontology group that is upregulated by HSF-1 in both a HS-dependent and -independent manner. The *C. elegans* cuticle is an exoskeletal structure that creates a barrier between the animal and the environment, provides body shape, and allows movement via attachment to muscle. Many of the genes in the cuticle structure category are collagens, structural proteins that form an extracellular matrix composing the exoskeleton, or cuticle, of the nematode. There are ~154 distinct collagen genes in *C. elegans*, and they are expressed in a tissue-specific fashion and at distinct temporal times [35]. Cuticle structure is controlled by enzymes involved in collagen processing, and the polymerization pattern is dictated by actin filaments that are organized in specific patterns around the body of the worm. In humans, collagens comprise about one-third of all expressed protein [36]. Aside from the structural role of collagens, these genes can also participate in signal transduction [37–39]. Collagen genes were recently found to be upregulated by SKN-1, the *C. elegans* oxidative stress-responsive transcription factor [40]. In future work, it will be interesting to test whether collagen can act to relay signals to stress-specific transcription factors including SKN-1 and HSF-1.

### Roles for HSF-1 in regulating metabolism and development in a HS-independent manner

Although HSF-1 has classically been studied as a transcription factor that responds to HS and cytoplasmic proteotoxic stress, HSF-1 has recently been gaining importance as a transcription factor that is involved in non-stress processes including development and metabolism [41, 42]. In mice, HSF family members have been documented to be involved in diverse developmental processes, including oogenesis, spermatogenesis, and corticogenesis [43]. HSF-1 in the worm has also been shown to be regulated by insulin/IGF-1, TGF-β, and cGMP signaling to control development [44]. The finding that mammalian HSF1 can be regulated by SIRT1, a deacetylase that is under metabolic control, provides evidence that HSF-1 and metabolism are linked [14]. This finding is also true for *C. elegans* HSF-1, as the SIRT1 homolog SIR-2.1 regulates the *C. elegans* HSR [45]. Additionally, the insulin-like signaling regulators DDL-1/2 have been linked to HSF-1 regulation [6]. Here, we show that under non-stress conditions, HSF-1 regulates a number of genes involved in developmental and metabolic processes. Therefore, our work further highlights the links between HSF-1 and these non-stress processes.

### Network analysis identifies a nuclear hormone receptor as a common link between processes regulated by HSF-1 upon HS

**Fig. 5:**
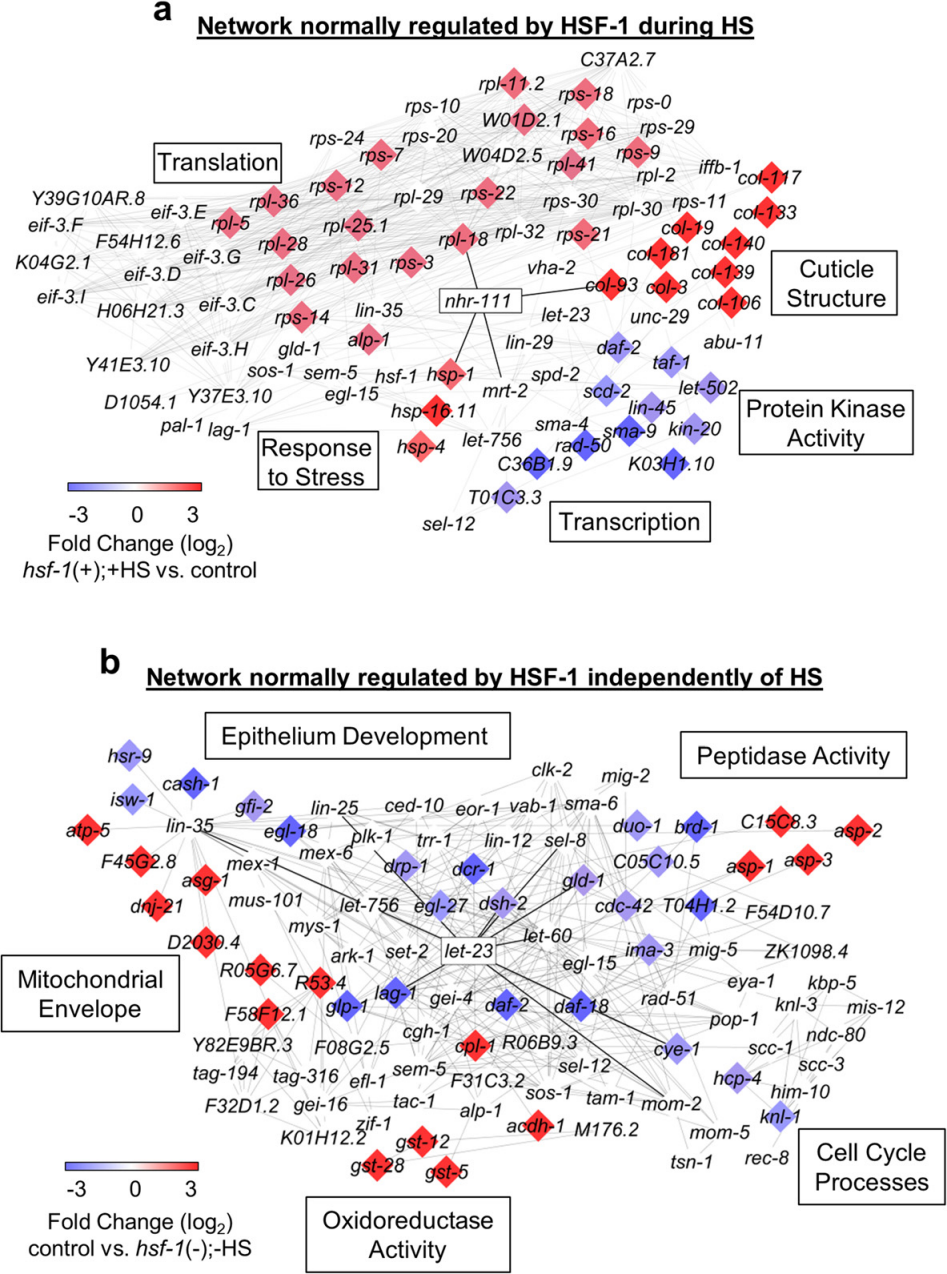
Network analyses of the top HSF-1-regulated processes. **a** Predicted network regulated by HSF-1 during HS**.** Genes associated with the top 5 induced and suppressed processes in Figs. 1c and 2c were used for analysis. **b** Predicted network regulated by HSF-1 independently of HS. Genes associated with the top 5 induced and suppressed processes in Figs. 3c and 4c were used for analysis. For **a** and **b**, the color of each gene corresponds to the degree of HSF-1 regulation of the corresponding transcript. Network analysis was done with MiMI using the Cytoscape platform. The uncolored genes were not affected by HSF-1 during or independently of HS in our dataset, but are neighbors shared by at least two genes that were affected in our dataset

To determine how the genes regulated by HSF-1 during HS may interact with each other, we performed network analysis using genes associated with the top GO-terms as determined by DAVID (Fig. 5). We used the MiMI plugin to integrate data from protein interaction databases (including gene ontology databases, MeSH, and PubMed) to allow for the creation of interaction networks using the network-building software Cytoscape. This analysis enabled us to identify interacting partners shared by at least two genes regulated by HSF-1 during HS. The transcripts induced by HSF-1 during HS are shown in red, while the transcripts downregulated by HSF-1 during HS are shown in blue, with the intensity of color correlating to the fold change. Genes that are not colored were not found to be affected by HSF-1 during HS in our dataset, but are neighbors shared by at least two genes that were affected.

We found network linkages between the processes of cuticle structure formation, translation, the response to stress, protein kinase activity, and transcription (Fig. 5a). Interestingly, the nuclear hormone receptor *nhr-111* is a common link between several of the HS-regulated processes that require HSF-1, including cuticle structure, translation, and the response to stress. Nuclear hormone receptors comprise a class of ligand-gated transcription factors that bind to small molecule metabolites such as fatty acids, vitamins, and steroids to directly regulate gene transcription [46]. They are thus well-poised to coordinate metabolism, development, reproduction, and homeostasis across diverse tissues. *nhr-111* is broadly expressed in *C. elegans* and is located in eight head neurons, the sensory PVD neurons in the posterior lateral body wall, the pharynx, the intestine, the dorsal peri-vulva region, and the somatic gonad precursor cells [47]. In future work, it will be interesting to test the role of *nhr-111* and other nuclear hormone receptors in the HSR, and to see if it can contribute towards the coordination of this response across tissues.

### Network analysis identifies a tyrosine kinase as a common link between various developmental processes regulated by HSF-1 independently of HS

To uncover *C. elegans* interaction networks associated with processes regulated by HSF-1 independently of HS, we performed network analysis using genes associated with the top GO-terms as determined by DAVID (Fig. 5b). Network linkages were found for genes involved in the mitochondrial envelope, peptidase activity, oxidoreductase activity, cell cycle processes, and epithelium development. We found that *let-23,* an epidermal growth factor receptor tyrosine kinase (EGF-RTK) [48], is predicted to interact with many of these transcripts. The EGF pathway in *C. elegans* has been linked to multiple developmental pathways [49]. This RTK may thus allow for signaling to HSF-1 during non-stress conditions to modulate developmental gene expression. Interestingly, HSF1 null mouse embryonic fibroblasts are defective in both basal and EGF-induced cell migration [50], so the link between HSF-1 and the EGF signaling pathways may be conserved across species. It will thus be worthwhile in future work to test for the involvement of *let-23* in the regulation of HSF-1 activity in *C. elegans*.

### HSF-1 impacts aging-regulated gene expression

**Fig. 6:**
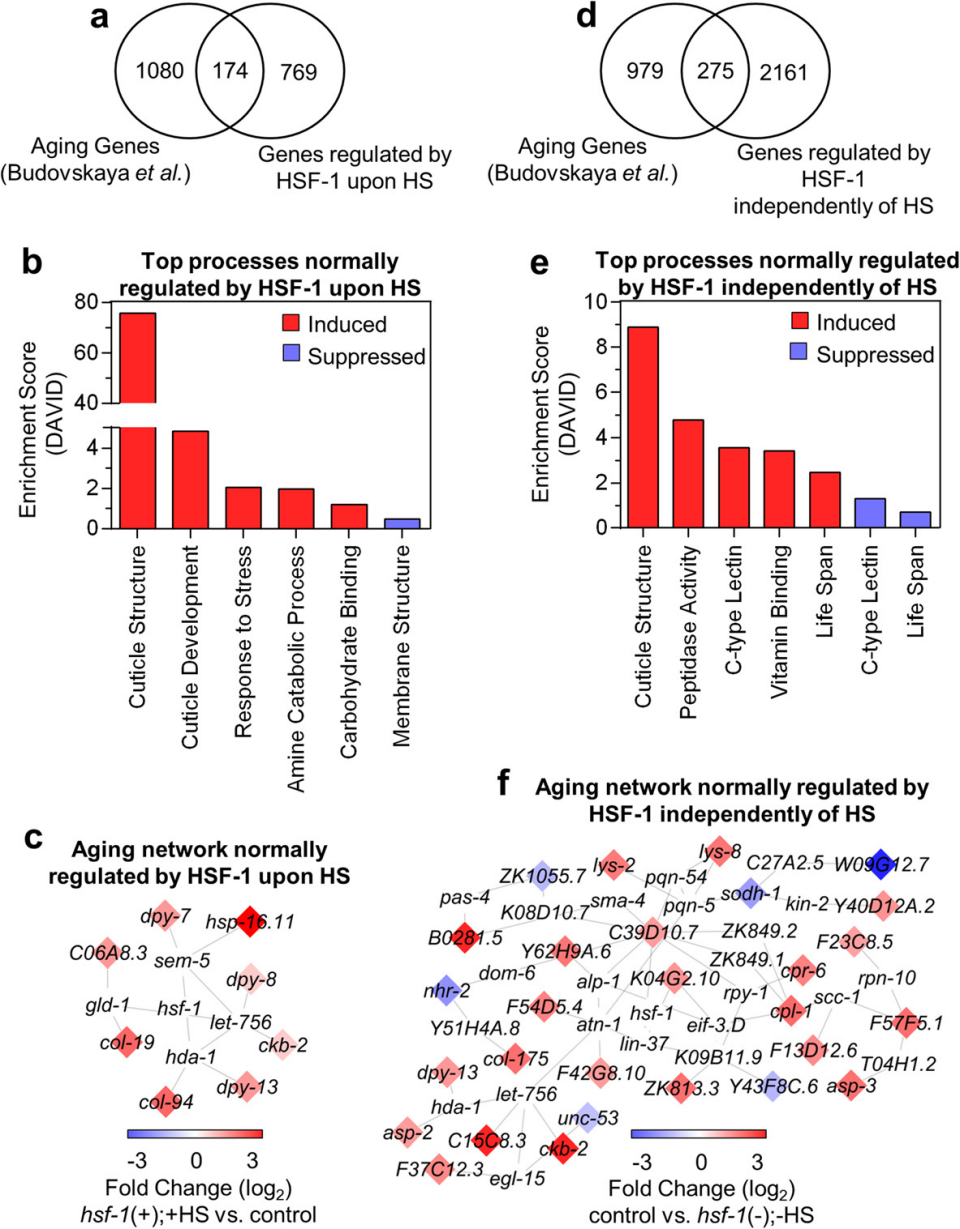
Age-regulated genes controlled by HSF-1. **a** The Venn diagram shows the overlap among genes that are differentially expressed during aging and regulated by HSF-1 during HS. The Venn diagram was made using genes previously found to be regulated during aging by *Budovskaya et al.* compared to genes we found to be regulated by HSF-1 during HS. **b** Cellular processes affected by aging and HSF-1 during HS. Genes shared between the aging dataset and HSF-1-regulated HS-dependent dataset from (**a**) were analyzed with DAVID and the Gene Ontology terms are listed in order of decreasing enrichment. **c** Network analysis of the genes regulated by aging and HSF-1 during HS. Network analysis was done with MiMI using the Cytoscape platform and the transcripts shared between data-sets (see Additional file 6: Table S5). The color of each transcript corresponds to the degree of HSF-1 regulation. Genes that are not colored were not affected by HSF-1 our dataset, but are neighbors shared by at least two genes that were affected in our dataset. **d** The Venn diagram shows the overlap among genes that are differentially expressed during aging and regulated by HSF-1 independently of HS. The Venn diagram was made using genes previously found to be regulated during aging by *Budovskaya et al.* compared to genes we found to be regulated by HSF-1 independently of HS. **e** Cellular processes affected by aging and HSF-1 independently of HS. Genes shared between the aging dataset and HSF-1-regulated HS-independent dataset from (**c**) were analyzed with DAVID and the Gene Ontology terms are listed in order of decreasing enrichment. **f** Network analysis of the genes regulated by aging and HSF-1 independently of HS. Network analysis was done with MiMI using the Cytoscape platform and the transcripts shared between data-sets (see Additional file 7: Table S6). The color of each transcript corresponds to the degree of HSF-1 regulation. Genes that are not colored were not affected by HSF-1 in our dataset, but are neighbors shared by at least two genes that were affected in our dataset

As HSF-1 has been implicated to play an important role in both aging and disease [51], we analyzed the role of HSF-1 in regulating age-associated transcriptional changes in our data-sets (Fig. 6). By comparing the transcriptome profiles between young and old adult *C. elegans,* a previous study by Budovskaya *et al.* identified 1,254 genes to be differentially regulated upon worm aging [52]. A Venn diagram comparison of these 1,254 age-regulated genes with those that we found to be regulated by HSF-1 during HS shows that 174 aging-associated genes overlapped with our dataset (Fig. 6a). A complete list of genes shared between both data-sets is also provided (see Additional file 6: Table S5). The functional processes regulated by this overlapping gene set were then determined via DAVID analysis. Cuticle structure was the largest functional category, with an enrichment score of 76 (Fig. 6b). Other gene categories, all with enrichment scores of 5 or lower, include cuticle development, the response to stress, amine catabolic processes, carbohydrate binding, and membrane structure. Network analysis performed on the 174 overlapping genes shows that only a small subset of these genes are predicted to interact with each other (Fig. 6c).

Next, a comparison between aging-associated genes and those we found to be regulated by HSF-1 independently of HS was done. A Venn diagram shows that 275 aging-associated genes overlapped with our dataset (Fig. 6d). A complete list of genes shared between both data-sets is also provided (see Additional file 7: Table S6). The functional processes regulated by this gene set was then determined via DAVID analysis. Cuticle structure was again the largest category, with an enrichment score of 9. The other categories, with enrichment scores of 5 or lower, include peptidase activity, C-type lectin, vitamin binding, and lifespan-associated processes (Fig. 6e). The transcriptional impact of these HSF-1 and aging-regulated genes that are independent of HS was then determined by network generation (Fig. 6f). Interestingly, the interaction network of aging-associated genes regulated by HSF-1 independently of HS is four-fold larger than the network of aging genes that depend on HS. We thus conclude that HSF-1 may have a role in impacting longevity that can be separated from its role in stress responses.

### HSF-1 regulates collagen genes which may affect the aging process

It is interesting that cuticle structure genes constitute the largest overlap with aging-related genes. In humans, mutations in collagens lead to a large number of heritable human diseases such as osteoporosis and musculoskeletal diseases [53]. Collagens are long-lived proteins known to accumulate damage during aging, leading to a decline in tissue health [54]. Also, type I collagens become resistant to proteolysis upon age [55, 56], affecting their turnover. Interestingly, mice expressing cleavage-resistant type I collagen go through an accelerated aging process [57]. Thus, cellular aging can be affected by the state of the extracellular matrix in mammals.

Recently, collagen production and extracellular matrix remodeling were determined to be essential for longevity in *C. elegans*. Collagen may directly affect signaling processes associated with longevity in *C. elegans*, including signaling through SKN-1 [40, 58]. We note that HSF-1 was also recently shown to regulate cytoskeletal integrity in a process that can influence stress resistance and longevity in *C. elegans* [59]. Thus, the linkage of both the extracellular matrix and the cytoskeleton to HSF-1 may provide a mechanism by which HSF-1 promotes longevity.

## Conclusion

Next generation sequencing has allowed us to uncover highly varied roles for *C. elegans* HSF-1 in both HS-dependent and -independent mechanisms, including roles in the regulation of development, cytoprotection, metabolism, and aging (for a model, see Additional file 1: Figure S11). Network analyses show that under HS conditions, the nuclear hormone receptor NHR-111 may allow coordination of the HSF-1 response across tissues, while under basal conditions, the EGF receptor LET-23 may regulate a similar coordination. These findings warrant further studies in order to further understand the methods of cell non-autonomous signaling across tissues. A striking result shown here is that multiple genes involved in cuticle structure, including collagen genes, are enriched as HSF-1 targets in HS-dependent and -independent manners. As recent studies link collagen to cytoprotection and longevity, the regulation of collagen expression may be one method by which HSF-1 enhances lifespan. Harnessing the ability of HSF-1 to regulate collagen could thus have broad appeal in the treatment of diseases of aging.

## Methods

### *C. elegans* strains and maintenance

The wild-type N2 strain, p*hsp-70*(*C12C8.1*)::GFP [10], p*hsp-16.2*(*Y46H3A.3*)::GFP [60], and EQ73 (HSF-1::GFP) [6] strains were used in this study. Worms were maintained at 23 °C on standard NGM plates seeded with *Escherichia coli* OP50 [61–63]. A synchronous population of nematodes was obtained by standard 20 % hypochlorite treatment, and a 24 h rotation at 220 rpm in M9 buffer without food.

### RNA interference and heat shock conditions

Approximately 4,000 wild-type nematodes were synchronized and placed at the L1 larval stage onto standard NGM plates supplemented with 50 μg/mL ampicillin and 1 mM isopropyl-beta--thiogalactopyranoside seeded with either HT115 bacteria containing an empty plasmid (L4440, control), or sequence-verified gene-specific RNAi isolated from the Ahringer RNAi library [64]. RNAi bacteria were allowed to induce on the plates overnight at room-temperature. Synchronized animals developed on RNAi plates before being treated at the L4 stage with a 30 min 33 °C HS by submerging the plates into a water bath. The time and duration of HS was optimized for this experiment (see Additional file 1: Figure S1b-d). Worms were then collected for RNA extraction.

### Immunoblotting and quantification

Animals were harvested in Buffer C (20 mM HEPES pH 7.9, 25 % Glycerol, 0.42 M NaCl, 1.5 mM MgCl2, 0.2 mM EDTA, and 0.5 mM DTT) with the addition of Halt™ protease inhibitors (Pierce, cat# 78430). Protein was extracted by sonication with a Diagenode Bioruptor 300 for 10 min with 30 s pulses. Protein was quantified by Bradford assay, resolved on a 10 % SDS-PAGE gel, and transferred to a PVDF membrane. The blot was incubated with an α-GFP polyclonal antibody (Abcam, cat# ab290) at a 1:2500 dilution and with α-Actin (Amersham, cat# JLA20-C) at a 1:750 dilution. Quantification of band intensity was performed using ImageJ Software (v. 1.44; http://imagej.nih.gov/ij/).

### RNA preparation for RNA-seq

Total RNA was prepared using TRIzol® reagent (Ambion®, cat# 15596-026) by standard protocols, and then cleaned up on RNeasy columns (QIAgen, cat# 74104). RNA integrity analysis, sample preparation, and RNA-sequencing was performed at the Yale Center for Genome Analysis using the Illumina HiSeq 2000 sequencing system.

### RNA-seq data analysis

A quality-control analysis of raw RNA-seq reads was performed using the FastQC program [65]. Short reads were aligned to the *C. elegans* reference genome (ws200 release) using Bowtie software [66]. The program TopHat was used to discover transcript splicing junctions [67]. The program Cufflinks was chosen to assemble the aligned reads, estimate their abundance, and calculate the fragments per kilobase of exon per million fragments mapped (FPKM) values [68]. Transcripts that were differentially expressed in different conditions, compared to the *hsf-1*(+);-HS control, were determined with CuffDiff, which uses the Benjamini-Hochberg correction for multiple testing to obtain the q-value (the FDR-adjusted the *p*-value) [69]. The results were visualized with a dendogram using the program CummeRbund [69]. The RNA-seq data has been deposited in NCBI SRA database (Access ID: SRP078295).

### Volcano plot analysis

Volcano plots were made using GraphPad Prism Software (GraphPad Software, La Jolla California USA, http://www.graphpad.com), where the Y-axis represents the q-value (FDR-corrected *p*-value) after being adjusted to reflect a -log_10_ value, and the X-axis represents the log_2_-fold change of each mRNA after comparison to the *hsf-1*(+);-HS control.

### Venn diagram analysis

Venny 2.0 [70] was used to construct Venn diagrams with the significantly altered mRNAs for each condition (q-value < 0.05) as compared to the *hsf-1*(+);-HS control.

### Quantitative RT-PCR

qRT-PCR was performed to validate the top hits from our RNA-seq data. An aliquot of the RNA samples that were used for sequencing were reverse transcribed into cDNA using a High Capacity cDNA Reverse Transcription Kit (Applied Biosystems, cat# 4368814) according to the manufacturer’s instructions. cDNA was diluted to 50 ng/μl to be used as a template for qRT-PCR which was performed with the Step One Plus Real-time PCR system (Applied Biosystems) using iTaq™ Universal SYBR® Green Supermix (BioRad, cat# 172-5121) according to manufacturer’s instructions. Data analysis was performed according to standard calculations using the comparative Ct method [71]. Relative mRNA levels were normalized to *gapdh*, and calculated from two biological replicates and technical triplicates. Primer sequences are available upon request. Statistical analyses were carried out with GraphPad Prism Software (GraphPad Software, La Jolla California USA, http://www.graphpad.com) using ANOVA followed by the Bonferroni post-hoc test. Error bars are representative of standard deviation between independent biological replicates.

### Fluorescence microscopy

Animals were anesthetized with 10 mM Levamisole and photographed using an EVOS fluorescence microscope. Image processing was accomplished using Adobe Photoshop© (Adobe Systems Incorporated, San Jose, CA).

### Heat map generation

The heat maps were organized using Cluster 3, by organizing the genes into 3 clusters, using K-means and 100 runs, and the Euclidean distance similarity metric [72].

### Gene ontology analysis via DAVID

The Database for Annotation, Visualization, and Integrated Discovery (DAVID) was used to identify over-represented gene ontology terms using the Functional Annotation Clustering tool and a high classification stringency [73]. The enrichment score provided by DAVID takes into account the probability that the members of a gene cluster are present randomly in the gene list. The enrichment score determines biologically significant functional groups by using the *p*-values for a cluster of genes to determine the geometric mean of that cluster (in negative log scale), where if the geometric mean of the *p*-values = 1e^−10^, then the enrichment score would be 10.

### Network analysis with the Cytoscape platform

Network analysis was performed using the MiMI plugin for the Cytoscape platform [74]. The MiMI plugin integrates data from protein interaction databases including gene ontology databases, MeSH, and PubMed to allow the creation of interaction networks using the network-building software Cytoscape. Interacting partners shared by at least two mRNAs were identified and used to construct interaction pathways.

## Abbreviations

DAVID, Database for Annotation, Visualization, and Integrated Discovery; EGF-RTK, Epidermal Growth Factor Receptor Tyrosine Kinase; EV, Empty Vector; HS, Heat shock; HSF-1, Heat Shock Factor 1; HSP(s), Heat Shock Protein(s); HSR, Heat Shock Response; qRT-PCR, quantitative RT-PCR; RNAi, RNA interference; RNA-seq, RNA-sequencing.

## Additional files

Additional file 1:
**Figure S1.** Scheme and validation of experimental conditions for RNA-seq experiments. **Figure S2.** Dendogram clustering of the biological duplicates for each RNA-seq condition reveals conserved alignment between replicates. **Figure S3.** Scheme for RNA-seq data normalization. **Figure S4.** Volcano plots show the global expression profile for each RNA-seq condition relative to the control. **Figure S5.** Genes regulated by development and molting share a similar expression profile between each RNA-seq treatment condition. **Figure S6.** The Venn diagram shows the total number of genes that were found to be significantly altered for each of the indicated comparisons between samples. **Figure S7.** Validation of top RNA-seq hits for genes normally upregulated by HSF-1 during HS via qRT-PCR. **Figure S8.** Collagen genes may control tissue-specific regulation of the HSR. **Figure S9.** Validation of top RNA-seq hits for genes normally downregulated by HSF-1 during HS via qRT-PCR. **Figure S10.** Validation of the top RNA-seq hits for genes normally regulated by HSF1 independently of HS via qRT-PCR. **Figure S11.** A model for major HSF-1 regulated processes in HS-dependent and -independent mechanisms. (DOCX 2280 kb) Additional file 2: Table S1. Significantly altered transcripts from each experimental condition compared to the *hsf-1*(+);-HS control. (XLSX 600 kb) Additional file 3: Table S2. List of genes found to be regulated by HSF-1 in a HS-dependent and -independent manner via Venn diagram analysis. (XLSX 130 kb) Additional file 4: Table S3. DAVID output of processes enriched by HSF-1 in a HS-dependent manner. (XLSX 38 kb) Additional file 5: Table S4. DAVID output of processes enriched by HSF-1 in a HS-independent manner. (XLSX 61 kb) Additional file 6: Table S5. Aging-related genes regulated by HSF-1 in a HS-dependent manner. (XLSX 15 kb) Additional file 7: Table S6. Aging-related genes regulated by HSF-1 in a HS-independent manner. (XLSX 18 kb)
