# Supplementary material for: The genome-wide role of HSF-1 in the regulation of gene expression in Caenorhabditis elegans
Source: BMC Genomics. 2016 Aug 5;17:559. doi: 10.1186/s12864-016-2837-5 (PMC4975890; doi:10.1186/s12864-016-2837-5)
Supplement: Additional file 1: — Figure S1. Scheme and validation of experimental conditions for RNA-seq experiments. Figure S2. Dendogram clustering of the biological duplicates for each RNA-seq condition reveals conserved alignment between replicates. Figure S3. Scheme for RNA-seq data normalization. Figure S4. Volcano plots show the global expression profile for each RNA-seq condition relative to the control. Figure S5. Genes regulated by development and molting share a similar expression profile between each RNA-seq treatment condition. Figure S6. The Venn diagram shows the total number of genes that were found to be significantly altered for each of the indicated comparisons between samples. Figure S7. Validation of top RNA-seq hits for genes normally upregulated by HSF-1 during HS via qRT-PCR. Figure S8. Collagen genes may control tissue-specific regulation of the HSR. Figure S9. Validation of top RNA-seq hits for genes normally downregulated by HSF-1 during HS via qRT-PCR. Figure S10. Validation of the top RNA-seq hits for genes normally regulated by HSF1 independently of HS via qRT-PCR. Figure S11. A model for major HSF-1 regulated processes in HS-dependent and -independent mechanisms. (DOCX 2280 kb) [file 12864_2016_2837_MOESM1_ESM.docx]

**b**

*hsf-1*(-);+HS vs. control

*hsf-1*(+);+HS vs. control


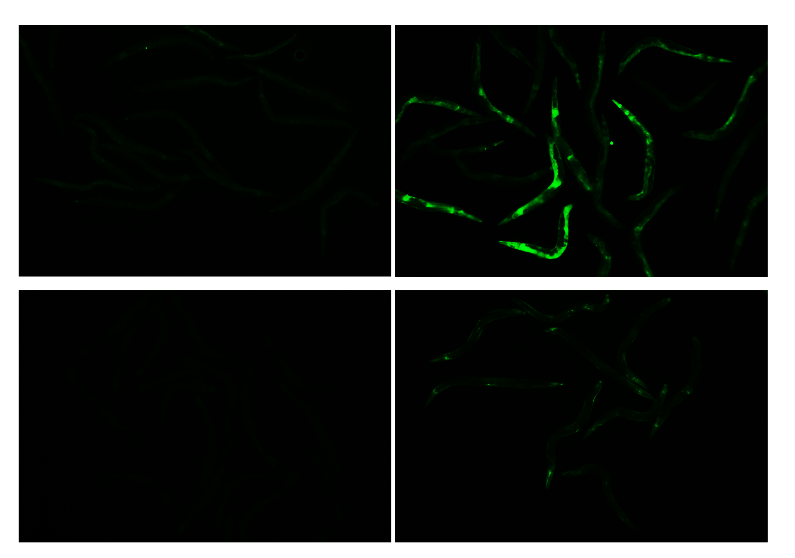


0.5 mm


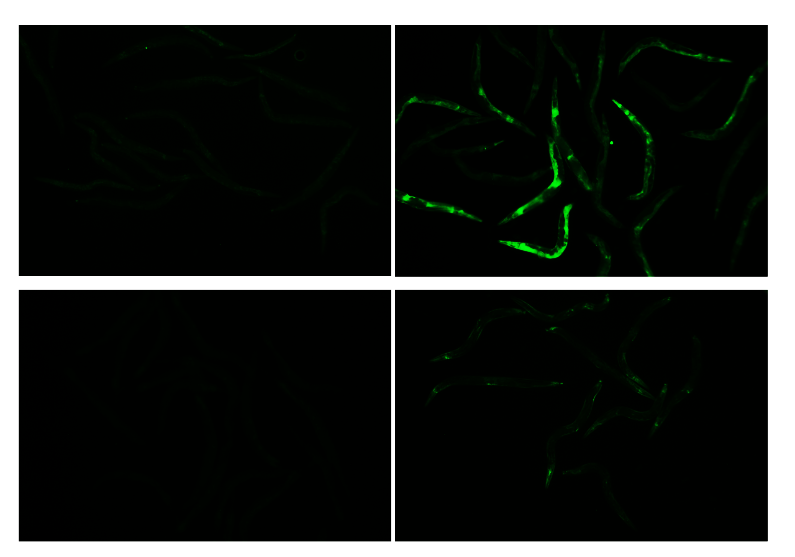


p*hsp-16.2* (*Y46H3A.3*)::GFP

control;+HS

control;-HS

*hsf-1*(-);-HS

*hsf-1*(-);+HS

HSF-1::GFP

α-Actin

α-GFP

control

*hsf-1*(-)


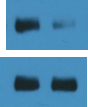

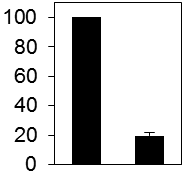


HSF-1::GFP

Band Intensity (%)

control

*hsf-1*(-)

***


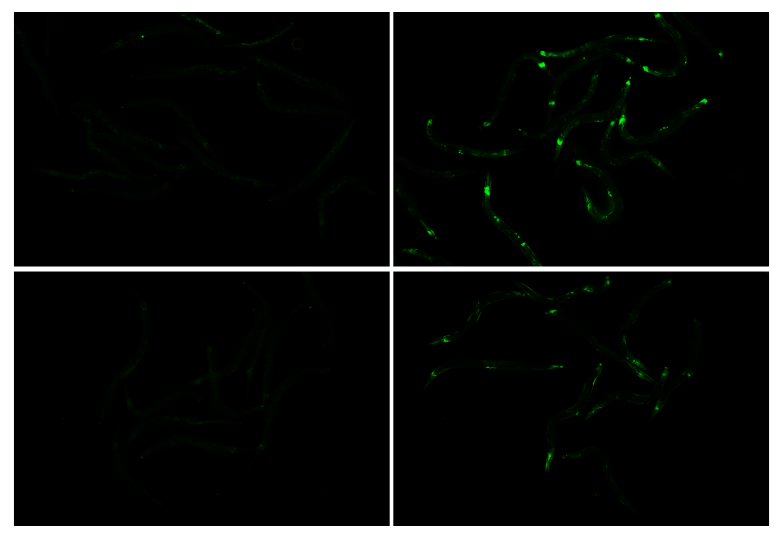


0.5 mm

p*hsp-70* (*C12C8.1*)::GFP

control;+HS

control;-HS

*hsf-1*(-);-HS

*hsf-1*(-);+HS

**a**

RNAi Treatment: L1🡪L4

EV RNAi (control)

*hsf-1* RNAi

*hsf-1*(+)

*hsf-1*(-)

-HS

+HS

Illumina Hi-Seq 2000

**Figure S1**

Scheme and validation of experimental conditions for RNA-seq experiments. **a** Experimental scheme for RNA-seq. RNA samples from wild-type L4 larval worms were generated, in two biological replicates, under the four indicated conditions, with “*hsf-1*(+)” referring to worms treated with empty vector (EV) RNAi control and “*hsf-1*(-)” referring to worms treated with *hsf-1* RNAi. “-HS” indicates that worms were left at growth temperature, while “+HS” indicates treatment with a 30 minute 33°C heat shock. RNA-sequencing was performed on the Illumina Hi-Seq 2000 platform. **b** *C12C8.1* (*hsp-70*) mRNA is robustly induced by a 33°C HS over a 15-120 minute time window. Synchronous wild-type (N2) L1 nematodes were treated with RNAi against *hsf-1* or with an empty vector (EV) control, indicated as *hsf-1*(-) or *hsf-1*(+), respectively. At the L4 larval stage, worms were either left at growth temperature or treated with a 33°C HS for the indicated times before RNA extraction. *hsp-70* mRNA levels were quantified with qRT-PCR and the results represent the average fold change from a set of biological duplicates and technical triplicates. Statistical significance was measured with One-Way ANOVA followed by Bonferroni’s post-test (***p<0.001). **c-d** *hsf-1* RNAi decreases HS-induced *hsp* promoter activity. Fluorescent images of synchronous p*hsp-70*::GFP or p*hsp-16.2*::GFP worms fed EV RNAi (control) or *hsf-1* RNAi from the L1 larval stage to the L4 larval stage prior to treatment with or without a 33°C 30 minute heat shock (HS) followed by a 12 hour recovery. **e-f** *hsf-1* RNAi decreases HSF-1 protein abundance. A transgenic worm strain containing GFP tagged HSF-1 under the control of its own endogenous promoter (HSF-1::GFP) was given the same RNAi feeding strategy in (c-d) prior to protein extraction and immunoblotting for GFP and actin. ImageJ was used to quantify the band intensity and determine the relative abundance of HSF-1.

**c**

**d**

**e**

**f**

1

0.050

0.055

0.060

0.065

0.070

0.075

0.080

*hsf-1*(-);+HS

1

2

*hsf-1*(+);-HS

Dissimilarity= 1-Correlation

Linkage Distance

1

2

2

*hsf-1*(-);-HS

1

2

*hsf-1*(+);+HS

0.085

**Figure S2**

Dendogram clustering of the biological duplicates for each RNA-seq condition reveals conserved alignment between replicates. The dendogram was generated with the program CummeRbund to provide insight into the relationships between different conditions. Significant differentially expressed genes used to draw the dendrograms are based on Jensen-Shannon distances.

*hsf-1*(-);-HS

*hsf-1*(+);-HS

(control)

*hsf-1*(+);+HS

*hsf-1*(-);+HS

**Data Normalization**

(1)

(2)

(3)

**Figure S3**

Scheme for RNA-seq data normalization. Each treatment condition was compared relative to the *hsf-1*(+);-HS control in order to determine fold changes in mRNA expression.

**b**

**a**


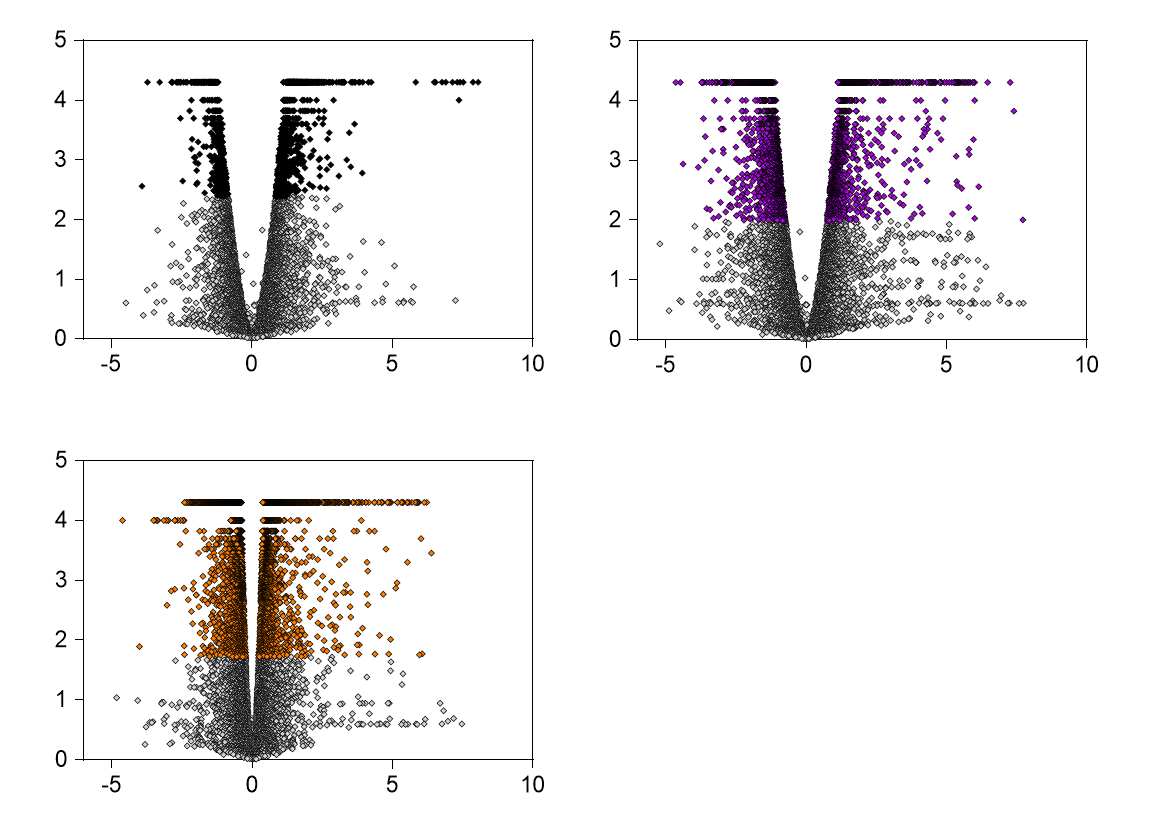


**Figure S4**

Volcano plots show the global expression profile for each RNA-seq condition relative to the control. **a** Volcano plot for *hsf-1*(+);+HS vs. the *hsf-1*(+);-HS control. Significantly altered genes (q-value<0.05), including several highly induced *hsp* genes are indicated in grey. **b** Volcano plot for *hsf-1*(-);-HS vs. *hsf-1*(+);-HS control. Significantly altered genes (q-value<0.05), including the vitellogenin genes *vit-1, vit-3, vit-4* and *vit-5*, are indicated. **c** Volcano plot for *hsf-1*(-);+HS vs. *hsf-1*(+);-HS control. Significantly altered genes (q-value<0.05), including the vitellogenin genes *vit-1, vit-3, vit-4* and *vit-5*, are indicated. For all, the q-value is the FDR-adjusted p-value of the test statistic, as determined by the Benjamini-Hochberg correction for multiple testing.

-q-value (log_10_)

Fold Change (log_2_)

*hsf-1*(-);-HS vs. control

-q-value (log_10_)

Fold Change (log_2_)

*hsf-1*(+);+HS vs. control

-q-value (log_10_)

Fold Change (log_2_)

*hsf-1*(-);+HS vs. control

*vit-1, vit-3, vit-4, vit-5*

*vit-1, vit-3, vit-4, vit-5*

*hsps*

**c**


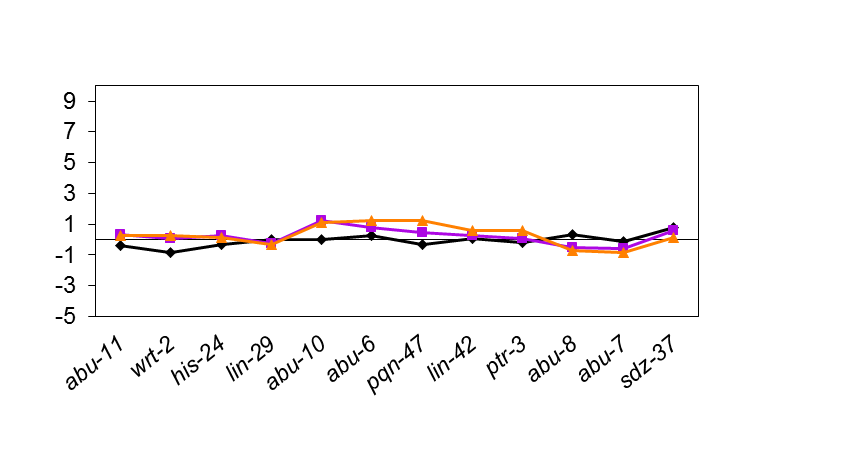


*hsf-1*(-);-HS vs. control

*hsf-1*(+);+HS vs. control

*hsf-1*(-);+HS vs. control

**Developmentally regulated/Molting genes**

Fold Change

(log_2_)

**Figure S5**

Genes regulated by development and molting share a similar expression profile between each RNA-seq treatment condition. The log_2_ fold change from our RNA-seq data for the indicated transcripts that are known to oscillate during development shows that no significant expression profile changes occur between datasets, indicating that treatment with *hsf-1* RNAi and/or HS did not affect the synchronicity of worms between our treatment conditions.


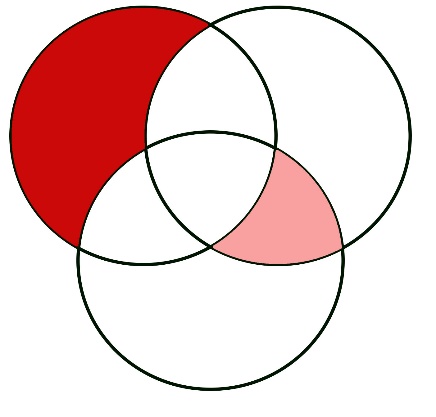


**Total Altered Genes**

q-value<0.05

(1) *hsf-1*(+);+HS

vs.

control

(2) *hsf-1*(-);-HS

vs.

control

(3) *hsf-1*(-);+HS

vs.

control

3223

231

658

4

59

2436

942

*Regulated by hsf-1 independently of HS*

*Regulated by hsf-1 upon HS*

**Figure S6**

The Venn diagram shows the total number of genes that were found to be significantly altered (q-value<0.05) for each of the indicated comparisons between samples. The q-value is the FDR-adjusted p-value of the test statistic, as determined by the Benjamini-Hochberg correction for multiple testing. The red area of the Venn diagram indicates genes that are regulated by HSF-1 upon HS, whereas the pink portion of the Venn diagram indicates genes that are regulated by HSF-1 independently of HS.

**a**

**b**

**Top genes that are normally upregulated by HSF-1 in response to HS**

**a**

control vs. *hsf-1*(-);-HS

*hsf-1*(+);+HS vs. control

control vs. *hsf-1*(-);+HS


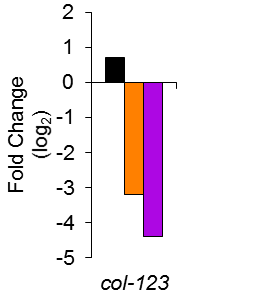


**RNA-seq**


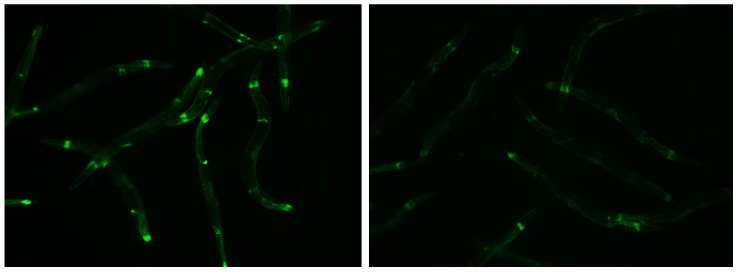


control;+HS

*col-123*(-);+HS


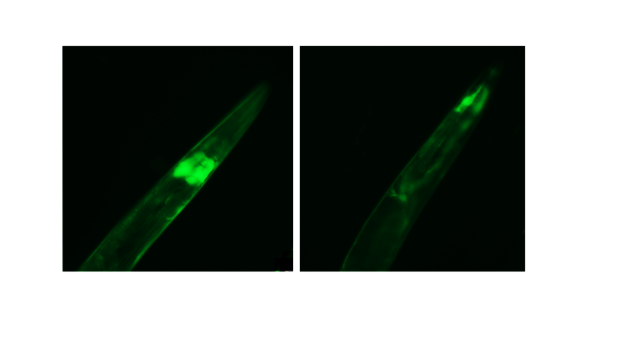


control;+HS

*col-123*(-);+HS

p*C12C8.1*(*hsp-70*)::GFP

p*C12C8.1*(*hsp-70*)::GFP

0.1 mm

0.5 mm

**b**

**c**

**Figure S8**

Collagen genes may control tissue specific regulation of the HSR. To verify that the presence of cuticle collagen genes in our dataset was not due to developmental timing differences between our treatment conditions, we tested the effects of *col-123* on induction of the HSR. **a** The log_2_ fold change of *col-123* mRNA expression based on RNA-seq data between each treatment condition. **b** Fluorescent images of synchronous p*hsp-70*::GFP worms fed EV RNAi (control) or *col-123* RNAi [*col-123*(-)] from the L1 larval stage to the L4 larval stage prior to treatment with or without a 33°C 30 minute heat shock (HS) followed by a 12 hour recovery. **c** The anterior of p*hsp-70*::GFP worms given the same treatment conditions as in (**b**) shows a decrease and shift in *hsp-70* promoter activity in response to treatment with *col-123* RNAi as compared to the control.

**Figure S7**

Validation of top RNA-seq hits for genes normally upregulated by HSF-1 during HS via qRT-PCR. **a** Fold changes of a subset of the top 15 upregulated genes as measured by RNA-seq analysis. **b** Validation of the same genes in (**a**) as measured by qRT-PCR analysis.

*hsf-1*(+);+HS vs. control

*hsf-1*(-);+HS vs. control

**b**

**Top genes that are normally downregulated by HSF-1 in response to HS**

*hsf-1*(+);+HS vs. control

*hsf-1*(-);+HS vs. control

**a**

**Figure S9**

Validation of top RNA-seq hits for genes normally downregulated by HSF-1 during HS via qRT-PCR. **a** Fold changes of a subset of the top 15 downregulated genes as measured by RNA-seq analysis. **b** Validation of the same genes in (**b**) as measured by qRT-PCR analysis.

control vs.

*hsf-1*(-);+HS

control vs.

*hsf-1*(-);-HS

**a**

**b**

**c**

**d**

**Top genes that are normally upregulated by HSF-1 independently of HS**

**Top genes that are normally downregulated by HSF-1 independently of HS**

**Figure S10**

Validation of the top RNA-seq hits for genes normally regulated by HSF-1 independently of HS via qRT-PCR. **a** Fold changes of a subset of the top 15 normally upregulated genes as measured by RNA-seq analysis. **b** Validation of the same genes in (**a**) as measured by qRT-PCR analysis. **c** Fold changes of a subset of the top 15 downregulated genes as measured by RNA-seq analysis. **d** Validation of the same genes in (**c**) as measured by qRT-PCR analysis. For (**a-d**) the data comparison was reversed [control vs. *hsf-1*(-);-HS or +HS] to obtain the fold change of these genes, in order to gain insight into the normal HSF-1-regulatory role of these HS-independent genes.


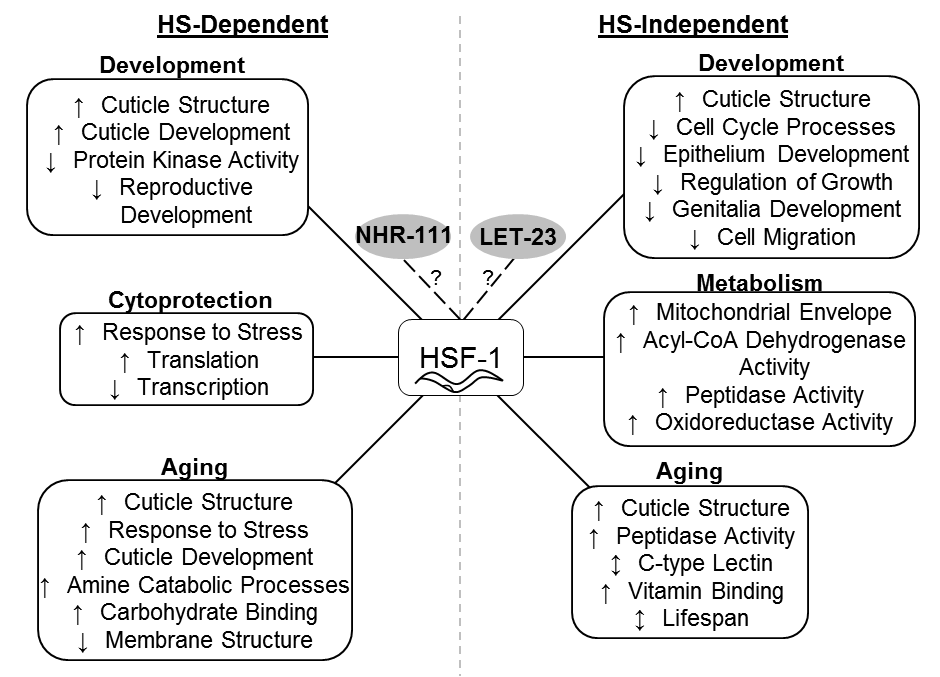


**Figure S11**

A model for major HSF-1 regulated processes in HS-dependent and -independent mechanisms. HSF-1 regulates a variety of processes during HS, but also has unique roles outside of the HSR. During HS, HSF-1 regulates development, cytoprotection, and aging, which may be regulated by the nuclear hormone receptor NHR-111. These processes are likely affected in order to promote survival to stress. The functions of HSF-1 outside of stress include roles in regulating development, metabolism, and aging, which may be regulated by a transmembrane tyrosine kinase LET-23. This highlights a role for HSF-1 in regulating both HS-dependent and –independent processes in *C. elegans*.
